# At What Stage of Neural Processing Does Cocaine Act to Boost Pursuit of Rewards?

**DOI:** 10.1371/journal.pone.0015081

**Published:** 2010-11-30

**Authors:** Giovanni Hernandez, Yannick-André Breton, Kent Conover, Peter Shizgal

**Affiliations:** 1 Department of Anatomy and Neurobiology, University of Maryland School of Medicine, Baltimore, Maryland, United States of America; 2 Center for Studies in Behavioral Neurobiology, Concordia University, Montréal, Québec, Canada; University of Minnesota, United States of America

## Abstract

Dopamine-containing neurons have been implicated in reward and decision making. One element of the supporting evidence is that cocaine, like other drugs that increase dopaminergic neurotransmission, powerfully potentiates reward seeking. We analyze this phenomenon from a novel perspective, introducing a new conceptual framework and new methodology for determining the stage(s) of neural processing at which drugs, lesions and physiological manipulations act to influence reward-seeking behavior. Cocaine strongly boosts the proclivity of rats to work for rewarding electrical brain stimulation. We show that the conventional conceptual framework and methods do not distinguish between three conflicting accounts of how the drug produces this effect: increased sensitivity of brain reward circuitry, increased gain, or decreased subjective reward costs. Sensitivity determines the stimulation strength required to produce a reward of a given intensity (a measure analogous to the *K_M_* of an enzyme) whereas gain determines the maximum intensity attainable (a measure analogous to the *v_max_* of an enzyme-catalyzed reaction). To distinguish sensitivity changes from the other determinants, we measured and modeled reward seeking as a function of both stimulation strength and opportunity cost. The principal effect of cocaine was a two-fourfold increase in willingness to pay for the electrical reward, an effect consistent with increased gain or decreased subjective cost. This finding challenges the long-standing view that cocaine increases the sensitivity of brain reward circuitry. We discuss the implications of the results and the analytic approach for theories of how dopaminergic neurons and other diffuse modulatory brain systems contribute to reward pursuit, and we explore the implications of the conceptual framework for the study of natural rewards, drug reward, and mood.

## Introduction

Long the province of mathematics, economics, psychology, and behavioral ecology, decision making is now the subject of burgeoning interest in neuroscience [1], [2], [3], [4], [5], [6], [7]. The objects of study range from nematodes to humans, and the levels of analysis extend from the molecular to the social group. Research initiatives aimed at unraveling the neural mechanisms underlying decision making are deployed along a broad front that encompasses perceptual [1], [8], cognitive [9], [10], affective [11], [12], [13], reward [2], [14], [15], and motor [16], [17] systems.

Among the crucial decisions that the nervous system must make on an ongoing basis is the allocation of time between competing behavioral goals. A rodent, for example, cannot simultaneously suckle her young and forage for the food and water required to sustain lactation [18]. Her choice of which activity to pursue at a given time depends on multiple variables, including costs, returns, risks, and the state of physiological stores. A major challenge for the neuroscientific analysis of decision making is to identify the neural circuitry that processes such variables, or effective proxies thereof, so as to generate biologically successful choices. A related challenge is to understand how, in the presence of the enriched, abundant, and inexpensive resources that modern technologies, economies and societies provide, such circuitry can generate choices leading to obesity, drug dependence, and unsustainable consumption.

The neuroscientific analysis of decision making is carried out in experimental paradigms designed to isolate and control key variables while providing meaningful measures of behavioral output and access to the underlying neural signals. One such paradigm entails delivery of electrical brain stimulation to laboratory animals as a reward for performance of instrumental actions such as pressing a lever or traversing an alley [19]. The effect that the subject works to reinstate, called “brain stimulation reward” (BSR), arises from an observable train of nerve impulses, triggered at a brain site chosen by the experimenter; the strength and timing of this volley can be controlled with great precision. In contrast to the repeated consumption of natural rewards such as food or water, repeated delivery of rewarding hypothalamic stimulation does not engender satiation [20], thus facilitating the collection of large datasets under stable conditions. These features made possible the detailed measurements reported here, which provide new information concerning the effect of cocaine on brain reward circuitry and reward-seeking behavior.

Dopamine neurons have been implicated strongly in decisions concerning reward-seeking [14], [21], [22], but the nature of their contribution remains a subject of lively debate. In early accounts, increases in dopaminergic neurotransmission were seen to boost the sensitivity of brain reward circuitry [23], [24]. On this view, cocaine, which blocks the dopamine transporter (DAT) and thus elevates extracellular dopamine levels, reduces the strength of the stimulus required to produce a rewarding effect of a given intensity (an action analogous to lowering the *K_M_* of an enzyme). According to an opposing view, changes in dopaminergic neurotransmission do not alter reward intensity but instead influence the proclivity to invest effort in pursuit of reward and/or the perceived magnitude of the invested effort [25], [26]. It has also been proposed that increased dopamine tone could boost the gain of brain reward circuitry [27], thus producing equal proportional changes in the rewarding impact of different stimuli (an effect orthogonal to a sensitivity change and analogous to increasing the *v_max_* of an enzyme-catalyzed reaction). As we demonstrate below, one cannot differentiate these views on the basis of data collected by conventional means because all three can produce equivalent effects when only a single independent variable is manipulated. However, a new method for measuring reward seeking [28], which entails varying both reward strength and reward cost, can distinguish sensitivity changes from changes in subjective effort costs or gain. That method is applied here to understand how cocaine alters pursuit of BSR. The results have implications for identifying the psychological processes that mediate the contribution of dopaminergic neurons to reward seeking and the stage(s) of neural processing at which this dopaminergic contribution is brought to bear. Dopaminergic neurons play a key role in the dependence-inducing effects of abused drugs [22]. Working out how these neurons influence the evaluation, selection and pursuit of rewards could well prove consequential to the development of effective treatments.

### The measurement of intracranial self-stimulation

As was the practice in most early studies of the effects of drugs on intracranial self-stimulation (ICSS) [29], [30], the effect of cocaine on this behavior was first measured by observing drug-induced changes in response rate [23]. Among the weaknesses of this one-dimensional measurement strategy are a) its inability to distinguish changes in the capacity to perform the rewarded behavior from changes in the strength of the rewarding effect [31], [32] and b) the dependence of the measured change in performance on the strength of the simulation.

**Figure 1:**
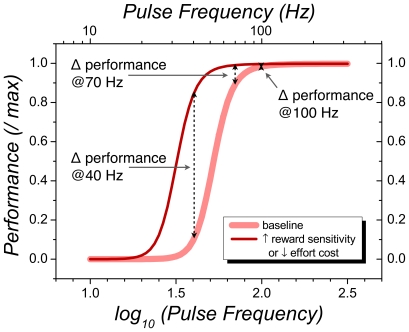
One- and two-dimensional approaches to measuring the effects of drugs on intracranial self-stimulation. The one-dimensional measurements are denoted by the dashed black lines wheres the two-dimensional measurements are denoted by the thin dark-red and thick pink curves.

The dashed black arrows in Figure **1** illustrate the weaknesses of the one-dimensional approach. Performance is measured at only a single value of stimulation strength (pulse frequency). Imagine that a large counterweight is affixed to the lever in the baseline condition, and thus the rat must exert considerable effort in order to earn a reward. With the pulse frequency set at 40 Hz, a drug can produce a large increase in performance (leftmost dashed black line) either by increasing the sensitivity of brain reward circuitry or by decreasing the perceived effort required to depress the lever [33], [34]. The one-dimensional data provide no way to determine which explanation is correct. Moreover, the size of the effects produced by the drug depends on the chosen value of stimulation strength. Increasing the pulse frequency to 70 Hz greatly reduces the observed change in performance, and increasing the pulse frequency to 100 Hz eliminates it.

To circumvent the deficiencies of the one-dimensional approach, a two-dimensional, “curve-shift” measurement strategy was introduced [35], [36], [37]. Performance is measured over a range of stimulation strengths, as depicted by the two colored curves in Figure 1. The effect of the drug is indexed by lateral displacement of the curve obtained under the influence of the drug (thin dark-red line) from the curve obtained under the drug-free (“baseline”) condition (thick pink line). Given that two curves are parallel, the size of the shift is independent of the level of performance chosen as the behavioral criterion. Nonetheless changes in either the energetic requirements of the rewarded response (effort cost) [33], [34] or the time required to perform it (opportunity cost) [38] can produce curve shifts indistinguishable from those due to changes in the sensitivity of brain reward circuitry. Arvanitogiannis and Shizgal [28] demonstrated that this ambiguity can be reduced by introducing a second independent variable, thus generalizing the measurement approach to three dimensions. In the current extension of their method, reward-seeking is measured as a function of both stimulation strength and opportunity cost, and the proportion of trial time that the rat allocates to pursuit of BSR (time allocation) serves as the measure of performance.

**Figure 2:**
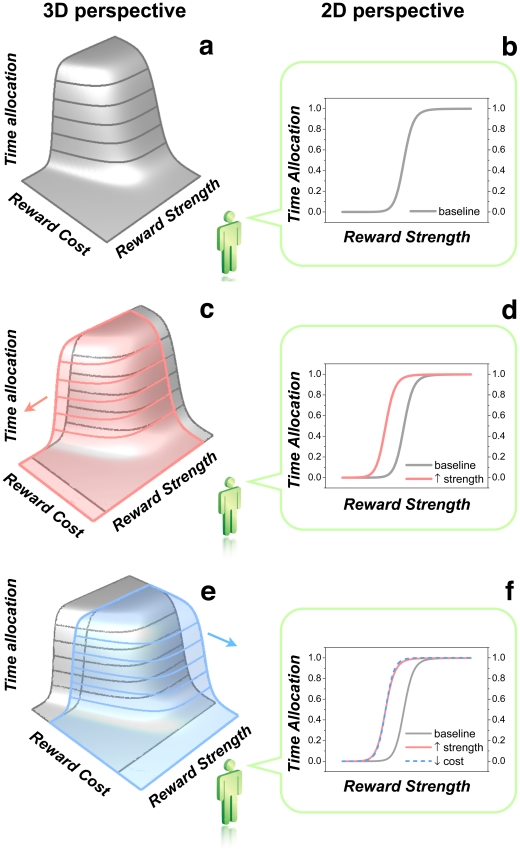
Shifts distinguishable in the 3D “reward-mountain” representation (left column) are ambiguous in the 2D “curve-shift” representation (right column). The little green figure (Shutterstock Images LLC) facing the reward-strength axis perceives the world in 2D. Thus, the price axis is invisible to him, and he sees the 3D structure as a 2D silhouette. Panels **b,d,f** show the left outlines of the silhouettes perceived by the little green figure. In panel **f**, the dashed blue outline of the mountain shifted along the price axis (panel **e**) is superimposed on the solid pink outline of mountain shifted along the reward-strength axis (panel **c**). Note that although the pink and blue mountains have been shifted in orthogonal directions and their displacements are readily distinguished in the 3D representations on the left, their 2D outlines (panel **f**) are virtually identical and could not be distinguished in any real experiment.

Figure **2** illustrates how the method of Arvanitogiannis and Shizgal adds crucial information to that provided by the curve-shift method. Panel **a** shows the three-dimensional (3D) structure produced by measuring time allocation as a function of both stimulation strength (pulse frequency) and opportunity cost (“price”). Time allocation is high when the stimulation is strong and inexpensive, and it declines as stimulation strength is decreased and/or the price is increased. The 3D structure is dubbed the “reward mountain.” The little green figure perceives the world only in two dimensions. Thus, from his vantage point, facing the reward-strength axis, the mountain is collapsed onto its two-dimensional (2D) silhouette in the plane defined by time allocation and reward strength; the left outline of the 2D silhouette is shown as a gray curve in panel **b**. Panel **c** depicts the effect of a drug that increases the sensitivity of the circuitry responsible for BSR; the stimulation strength (e.g., pulse frequency) required to produce a reward of a given intensity has been reduced by the drug, and thus the mountain (shown in pink) has shifted leftward along the reward-strength axis. The silhouette of the shifted 3D structure is shown as a pink curve in panel **d**. Panel **e** depicts the effect of a drug that decreases subjective effort costs and thus shifts the mountain (shown in blue) rightward along the price axis. The silhouette of the shifted 3D structure is shown as a light blue, dashed curve in panel **f**, superimposed on the pink curve showing the silhouette of the mountain that shifted along the reward-strength axis. Although the two shifts are orthogonal and can be distinguished clearly in the 3D representations in panels **c,e,** their two dimensional (2D) projections (in panel **f**) are virtually identical. Thus, 2D curve shifts are inherently ambiguous. On the basis of an observed displacement in a 2D representation (such as those in panels **d,f**), one cannot determine the direction in which the corresponding 3D structure has moved. (Movie S1 and Movie S2 provide additional demonstrations of the ambiguity of 2D representations.)

### The reward-mountain model

**Figure 3:**
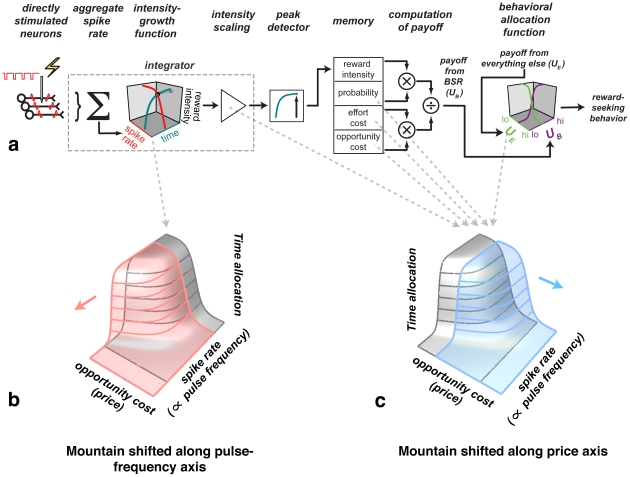
Neural signals and computations that translate the stimulation-induced volley of action potentials into reward-seeking behavior. **a**) The “minimal model.” **b,c**) Shifts of the reward mountain produced by drug-induced changes at different stages of neural processing that contribute to reward seeking. Drug actions prior to the output of the intensity-growth function shift the mountain along the pulse-frequency axis (**b**) whereas actions beyond the output of the intensity-growth function shift the mountain along the opportunity-cost (“price”) axis (**c**).

The three-dimensional (3D) method proposed by Arvanitogiannis and Shizgal [28] is grounded in a “minimal model” that ties behavioral allocation to the intensity and costs of reward (Fig. **3a**), providing a rigorous framework for interpreting drug-induced shifts in the reward mountain. The model is minimal in the sense that it is built exclusively from components essential to account for fundamental properties of ICSS; it is unlikely that any successful alternative would be significantly simpler. The model extends a proposal by Gallistel [39]; a formal derivation is provided in Text S1.

Signal flow in Figure **3a** proceeds from left to right. The behaviorally relevant aspect of the induced neural activity is the aggregate firing (“spike”) rate of the directly stimulated neurons (Σ) [39], [40], [41]. The aggregate firing rate is directly proportional to the pulse frequency, provided that the pulse frequency is sufficiently low for each directly stimulated neuron to fire once per pulse. Reward intensity, a proxy for reward quality, grows non-linearly as a conjoint function of the aggregate spike rate and the time during which the stimulation is applied [41], [42], approaching asymptote as either the induced spike rate or the duration of the stimulation train is raised to high values. The scaling of the output of the intensity-growth function is represented by the triangular amplifier symbol; the separation of the output scaling from the parameters controlling the shape and location of the intensity-growth function provides a graphical representation that parallels the distinction drawn below between the sensitivity and gain of the BSR substrate.

Evaluation of BSR manifests a property called “duration neglect”; once the duration of a stimulation train exceeds a critical duration, further increases in duration do not add to the subjective valuation of the train [42]. A parsimonious way to account for this property is to pass the output of the intensity-growth function through a peak detector [39], [42] en route to memory so that only the maximum value registered during the stimulation train is stored.

The behavior of the self-stimulating subject is a function of the strength and cost of stimulation received in the past. Thus, the minimal model must include a mnemonic component [43]. The stored values shown in Figure **3a** are subjective estimates of the peak reward intensity, reward probability (the likelihood that a stimulation train will be delivered when the response requirement is met), effort cost (the rate of exertion required to meet the response requirement), and opportunity cost (the time required to meet the response requirement). In accord with theoretical accounts of operant performance [44], [45], [46], the stored values are combined in scalar fashion. We refer to the result of this scalar combination as the “payoff” from BSR. This quantity is compared to the payoff from alternate activities, such as grooming, resting and exploring, so as to determine the allocation of behavior between ICSS and the other activities that can be performed in the test cage (“everything else”) [47], [48], [49].

With the aid of the model, shifts in the position of the reward mountain can be related to different variables and stages of processing that contribute to reward seeking. Displacement along the pulse-frequency axis (Fig. **3b**) is caused by influences brought to bear prior to the output of the intensity-growth function, such as lesions that reduce the number of directly stimulated neurons, or drugs that alter their synaptic output. In contrast, the mountain is displaced along the price axis (Fig. **3c**) by influences brought to bear at later stages of the model, such as scalar changes in reward intensity (i.e., changes in the gain of the BSR substrate) or changes in subjective effort costs.

### Effects of cocaine on pursuit of BSR from the perspective of the reward-mountain model

If cocaine were to increase the release and/or persistence of neurotransmitter from neurons upstream from the intensity-growth function in Figure **3a**, fewer spikes would be required to produce a given level of reward intensity because of the increased impact of each spike. As shown in Figure **3b**, this would displace the reward mountain leftward along the pulse- frequency axis. This shift reflects a drug-induced increase in the sensitivity of the reward circuitry to the electrically induced volley of action potentials. In early studies of the role of catecholaminergic neurons in ICSS [23], [50], drug-induced changes in behavior were interpreted in terms of such alterations in sensitivity. However, as Figure 2 illustrates, sensitivity changes cannot be inferred unambiguously from 2D data; 3D data are required to distinguish lateral displacement of the intensity-growth function from alternate actions of the drug, such as alteration of gain or subjective effort cost, which would shift the mountain rightward along the opportunity-cost (“price”) axis (Fig. **3c**). By measuring performance as a function of both the strength and cost of BSR, the necessary 3D information was acquired in the present experiment, which is the first to apply the method of Arvanitogiannis and Shizgal [28] to studying the effects of drugs on reward seeking. Although some cocaine-induced increases in the sensitivity of the reward-generating circuitry were noted, increased gain and/or decreased subjective costs can account for the larger and more consistent effect of cocaine on reward-seeking: a two-fourfold increase in willingness to pay. These findings have important implications for competing theories of how dopaminergic neurons contribute to reward seeking.

## Results

### Histology

**Figure 4:**
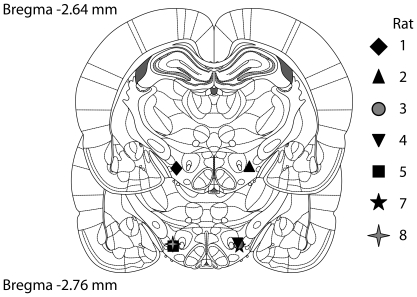
Location of the electrode tips.

As shown in Figure **4**, All of the electrode tips were located within the lateral hypothalamus, in the coronal planes corresponding to Plates 55 and 56 of the Paxinos and Watson atlas [51].

### Behavioral data

A reward was delivered when the cumulative time that the rat depressed a retractable lever reached a criterion duration (the opportunity cost or “price” of the stimulation). The cumulative hold time was frozen during periods when the lever was released and began incrementing again when the lever was next depressed. When the criterion was attained, the hold time was reset to zero. Stimulation strength was controlled by varying the pulse frequency.

#### Comparisons between variants of the 3D model and between alternate surface-fitting procedures

Resampling [52] was used to fit the 3D model to the behavioral data and to estimate confidence intervals surrounding the parameters of the best-fitting surface. Two variants of the 3D model were fit. The seven-parameter variant includes a term for the value of conditioned reward whereas this term is absent from the six-parameter variant (Text S1). Each variant was fit to the data in two ways. The “location-specific” method minimizes a bias that would otherwise cause the slopes of the fitted surface along the pulse-frequency and price axes to be underestimated whereas the “all-common” method reduces the number of parameters in the model and the uncertainty surrounding the parameter estimates (see Materials and Methods). The Akaike Information Criterion (AIC) [53] was used to determine which variant of the model and which fitting method was best.

#### The location-specific versus the all-common variants of the model

As shown in Table **1**, the location-specific method yielded the better AIC scores [53] for all 7 sets of vehicle data and for 4/7 sets of cocaine data (from Rats 2, 3,5, 8) obtained by sweeping the values of the price and pulse frequency. The location-specific method was also best, both in the saline and cocaine conditions, when applied to the data from Rat 4 that were obtained by means of random sampling of prices and pulse frequencies (Table **2**).

**Table 1:** AIC values, sweep sampling (most negative is best).

	Saline Condition				Cocaine Condition			
	6-parameter		7-parameter		6-parameter		7-parameter	
Rat	all-common	location-specific	all-common	location-specific	all-common	location-specific	all-common	location-specific
**1**	−2,049.5	**−2,133.7**	−2,053.1	−2,107.0	−828.3	−813.3	**−841.5**	−819.4
**2**	−4,541.0	**−4,767.7**	−4,558.5	−4,698.5	−885.6	−977.1	−1,023.6	**−1,055.3**
**3**	**−**3,350.6	**−3,456.9**	−3,396.2	−3,439.1	−791.4	−792.0	−841.8	**−911.8**
**4**	−1,628.2	−1,638.3	−1,626.1	**−1,638.7**	−1,139.0	−1,134.4	**−1,140.0**	−1,131.7
**5**	−1,683.0	−1,688.5	−1,693.9	**−1,695.9**	−1,318.9	−1,305.1	−1,319.8	**−1,321.2**
**7**	−2,396.3	**−2,483.1**	−2,397.7	−2,463.4	−1,357.5	−1,344.2	**−1,392.0**	−1,388.2
**8**	−1,738.6	**−1,836.5**	−1,742.5	−1,756.5	−925.7	**−951.6**	−923.6	−941.1

**Table 2:** AIC values, random sampling (most negative is best).

	AIC values, random sampling (most negative is best)							
	Saline Condition				Cocaine Condition			
	6-parameter		7-parameter		6-parameter		7-parameter	
**Rat**	all-common	location-specific	all-common	location-specific	all-common	location-specific	all-common	location-specific
**4**	−992.1	**−999.8**	−990.4	−987.7	−881.8	**−893.1**	−884.4	−892.3

#### The conditioned-reward model

The AIC score was also used to determine whether it was justified to add a parameter reflecting conditioned reward (*F_CR_*, Eqs. 8,9 in the Text S1). On this basis, it was determined that inclusion of the *F_CR_* parameter was justified in the fits to 6 of the 7 sets of data from the cocaine condition as well as in the fits to 2 sets of data from the saline condition obtained by sweeping the values of the price and pulse frequency (Table **1**); in one of the cases from the saline condition, the value of this parameter was too low to visibly alter the shape of the contour lines (not shown). The 6-parameter model fit best to the data obtained by means of random sampling of prices and pulse frequencies, both in the saline and cocaine conditions.

As shown in Tables **3** **,** **4**, the adjusted R^2^ values for the best-fitting surfaces ranged from 0.961 - 0.982 (saline) and from 0.919 - 0.97 (cocaine). Thus, the 3D surfaces fit the time-allocation data well.

**Table 3:** Goodness-of-fit, sweep sampling.

Goodness-of-fit, sweep sampling		
Rat	Adjusted R^2^, saline	Adjusted R^2^, cocaine
1	0.973	0.953
2	0.974	0.919
3	0.977	0.964
4	0.961	0.956
5	0.977	0.970
7	0.982	0.946
8	0.973	0.928

**Table 4:** Goodness-of-fit, random sampling.

Goodness-of-fit, random sampling		
Rat	Adjusted R^2^, saline	Adjusted R^2^, cocaine
4	0.944	0.924

#### Two-dimensional representation

**Figure 5:**
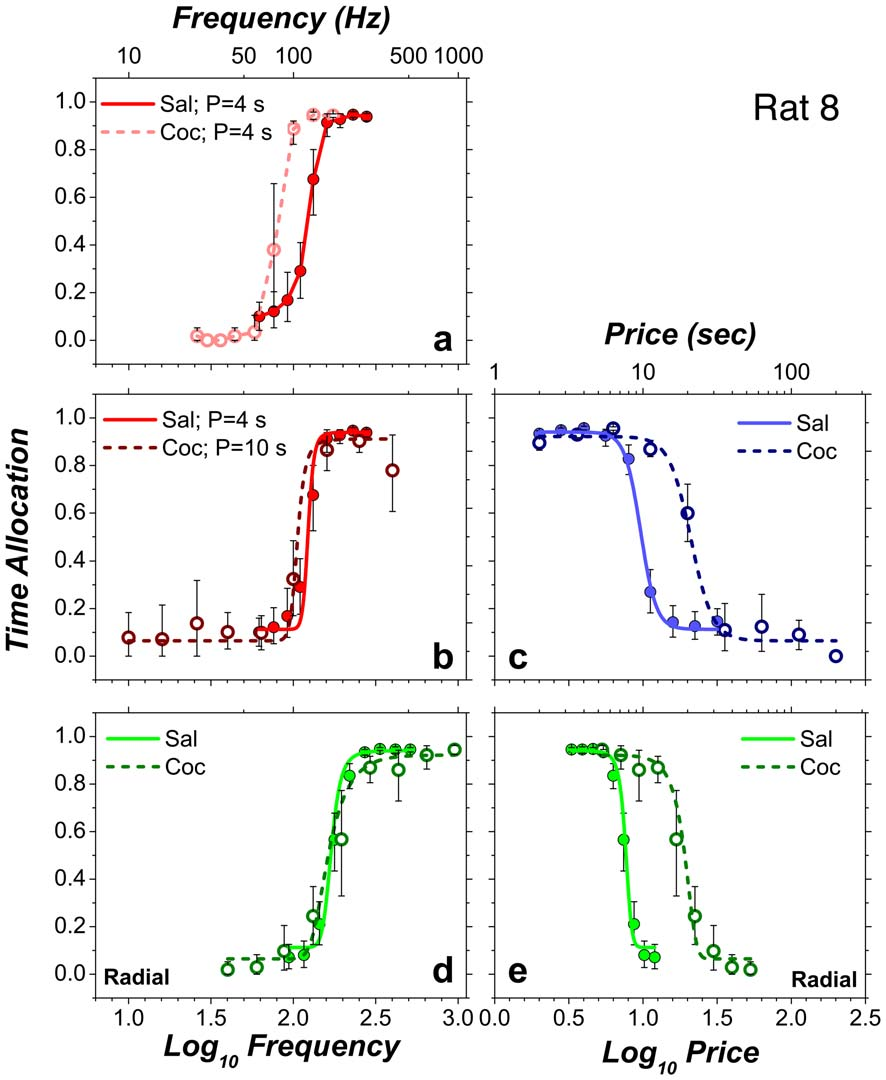
Two-dimensional representation of the results from Rat 8. Data from frequency sweeps are shown in shades of red, data from price sweeps in shades of blue, and data from radial sweeps in shades of green.

Figure **5** shows the results from one subject (Rat 8) in a two-dimensional (2D) format. The strength and/or price of the stimulation was varied sequentially (“swept”) from trial to trial. Panel **a** is analogous to the conventional representation of curve-shift data. The price of the stimulation was 4 s, and the pulse frequency was swept so as to drive time allocation from its maximal to its minimal value. Continuous subcutaneous infusion of cocaine (1.75 or 3.5 mg/kg/hour) shifted the frequency-sweep curve leftward, decreasing the strength of the stimulation required to induce the rat to allocate a given proportion of its time to pursuit of BSR. An increase in the price of the BSR from 4 to 10 s counteracted the effect of cocaine (panel **b**). Panel **c** shows the effect on reward pursuit of sweeping the price of a high pulse-frequency (400 Hz) stimulation train. When the price was low, the rat held down the lever almost the entire time it was extended, and thus, the proportion of time the lever was depressed (time allocation) was close to 1. As the price was increased, time allocation fell in a sigmoidal manner. Cocaine shifted the price-sweep curve rightward, increasing the time the rat was willing to invest in the pursuit of BSR. Panels **d,e** show the results obtained during “radial sweeps,” which were carried out by simultaneously increasing the price and decreasing the pulse frequency from trial to trial. Note that no shift is apparent when the radial-sweep data are projected along the pulse-frequency axis (panel **d**) but that a large shift is seen when they are projected along the price axis (panel **e**).

**Figure 6:**
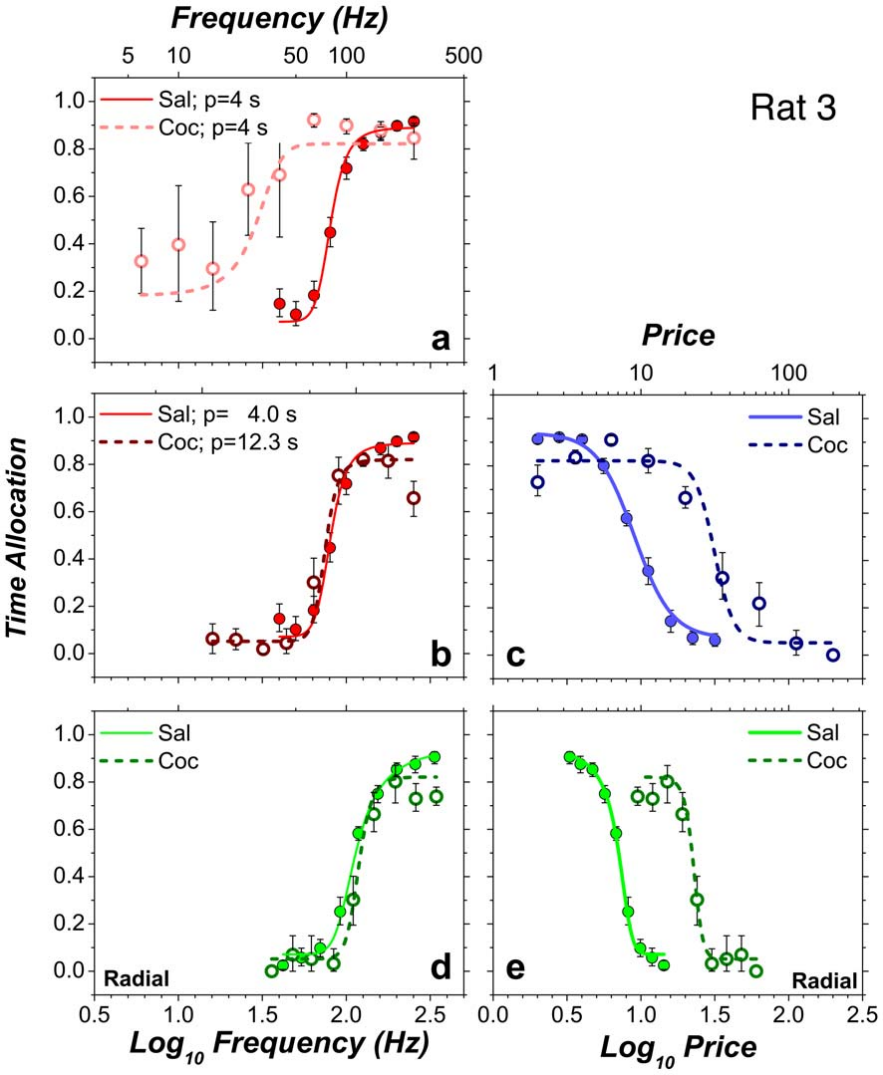
Two-dimensional representation of the results from Rat 3.

At low pulse frequencies, Rat 8 showed similar low allocation of time to reward pursuit during infusion of saline and cocaine. Four of the remaining subjects allocated substantially more time at low pulse frequencies during cocaine infusions than during saline infusions, particularly when the price was low, as illustrated by the results from Rat 3 (Fig. **6a**). Panel **b** shows that increasing the price at which the frequency sweep was conducted (from 4 to 12.3 s) not only nulled the effect of cocaine on the position of the psychometric curve but also eliminated the drug-induced boost in the allocation of time to pursuit of low-frequency stimulation trains.

#### Three-dimensional representation

**Figure 7:**
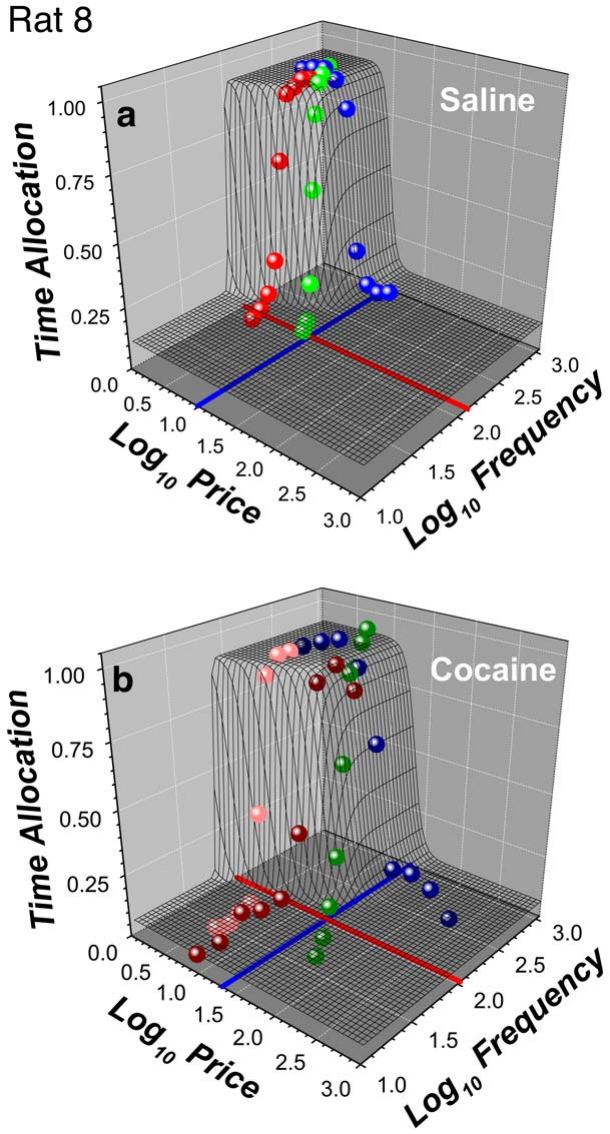
Three-dimensional view of the data for a single subject (Rat 8). **a**) Mean time allocation values for the saline condition along with the wire-mesh depiction of the 6-parameter surface fitted to them. The blue and red lines represent the values of the *P_e_*, and *F_hm_* parameters, which determine the position of the 3D structure along the price and frequency axes, respectively. **b**) 3D representation of the data from the cocaine condition. Data from frequency sweeps are shown in shades of red, data from price sweeps in shades of blue, and data from radial sweeps in shades of green.

The data from Figure **5** are re-plotted in a three dimensional (3D) view in panels **a,c** of Figure **7**, superimposed on a wire-mesh depiction of the surface (the “reward mountain”) obtained by fitting the model illustrated in Figure **3a** and described formally in Text S1.

**Figure 8:**
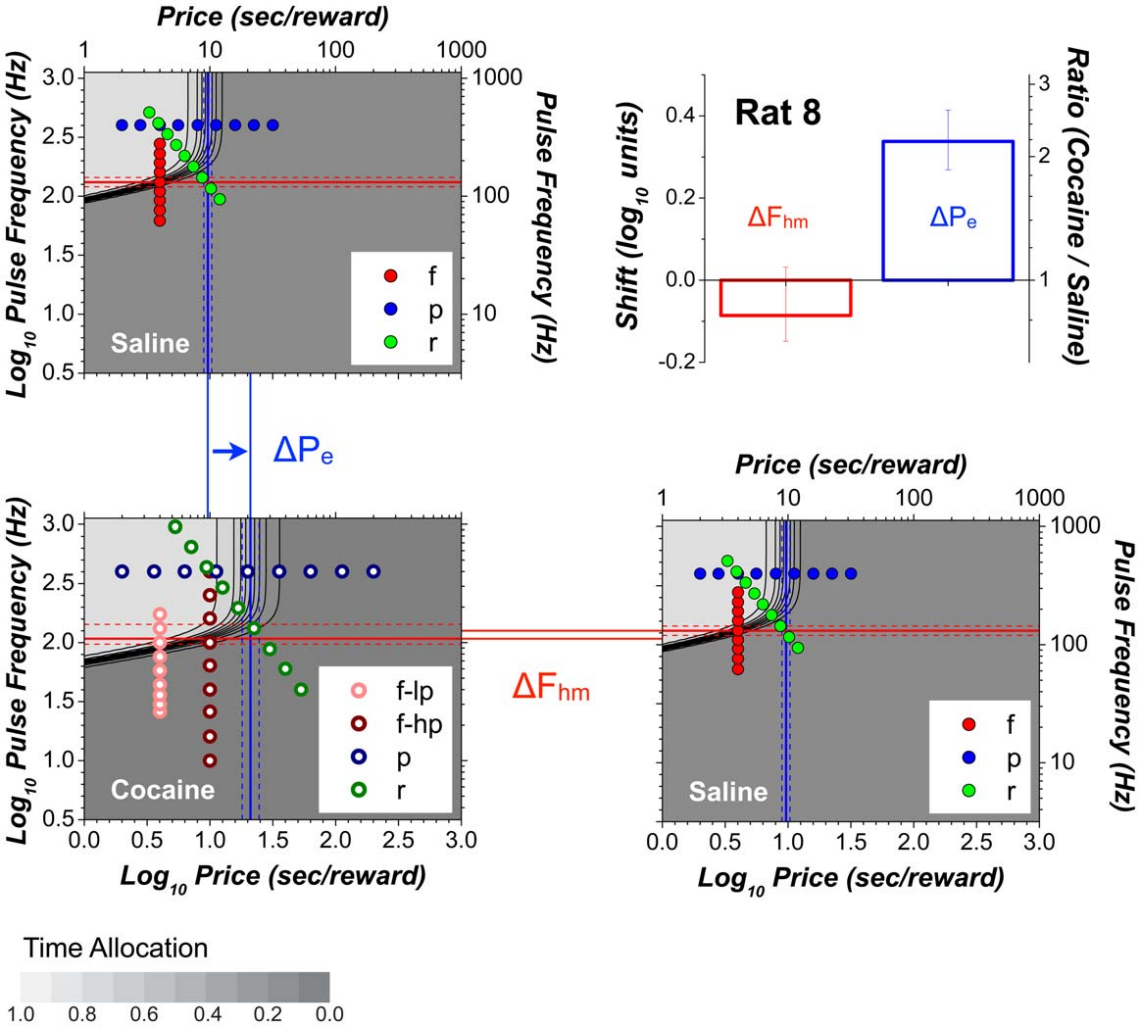
Shifts caused by cocaine in the position of the 3D structure fitted to the data from a single subject (Rat 8). The data from Figure **7** are re-plotted as contour graphs so as to isolate and highlight the drug-induced shifts of the mountain (Δ*P_e_*, Δ *F_hm_*) along the price and frequency axes. The magnitudes of the shifts are contrasted in the bar graph. Error bars in the bar graph and dashed lines on the contour graphs are 95% confidence intervals. Data from frequency sweeps (“f”) are shown in shades of red, data from price sweeps (“p”) in shades of blue, and data from radial sweeps (“r”) in shades of green. “lp” and “hp” designate data from frequency sweeps carried out at low and high prices, respectively.

The 3D information from Figure **7** is compressed into contour graphs in Figure **8** so as to highlight the drug-induced displacement of the 3D structure. The contour graph of the results obtained in the saline condition is shown twice, in the upper left and lower right, thus making clear the degree to which cocaine displaced the 3D structure along the pulse-frequency and price axes. The magnitudes of these movements (Δ*F_hm_*, Δ*P_e_*) are summarized in the bar graph in the upper right. Displacements are considered to be reliable statistically when zero falls outside the 95% confidence interval (error bars) surrounding the shift estimate.

**Figure 9:**
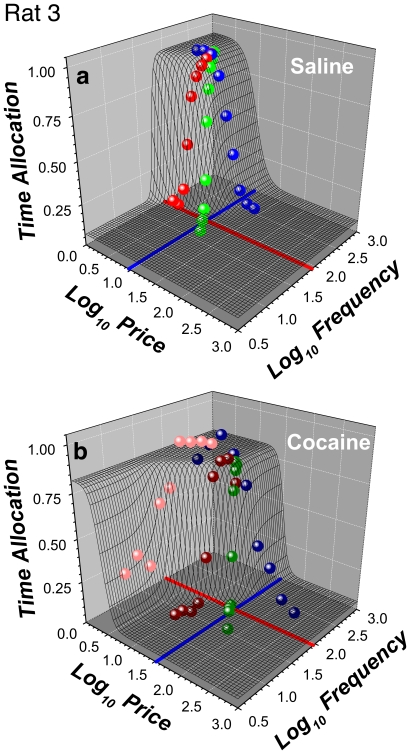
Three-dimensional views of the data from Rat 3. **a**) Mean time allocation values for the saline condition along with the wire-mesh depiction of the 6-parameter surface fitted to them. The blue and red lines represent the values of the *P_e_*, and *F_hm_* parameters, which determine the position of the 3D structure along the price and frequency axes, respectively. **b**) 3D representation of the data from the cocaine condition. Note the elevated time allocated to low-frequency trains under the influence of cocaine at low (pink spheres), but not high (dark red spheres), prices. The wire-mesh surface in **b**) describes the fit of the 7-parameter “conditioned-reward” model to the data.

Figures **9** **,** **10** provide plots analogous to those in Figures **7** **,** **8** but for a rat that showed elevated time allocation under the influence of cocaine over the low-frequency portion of the low-price frequency sweep.

**Figure 10:**
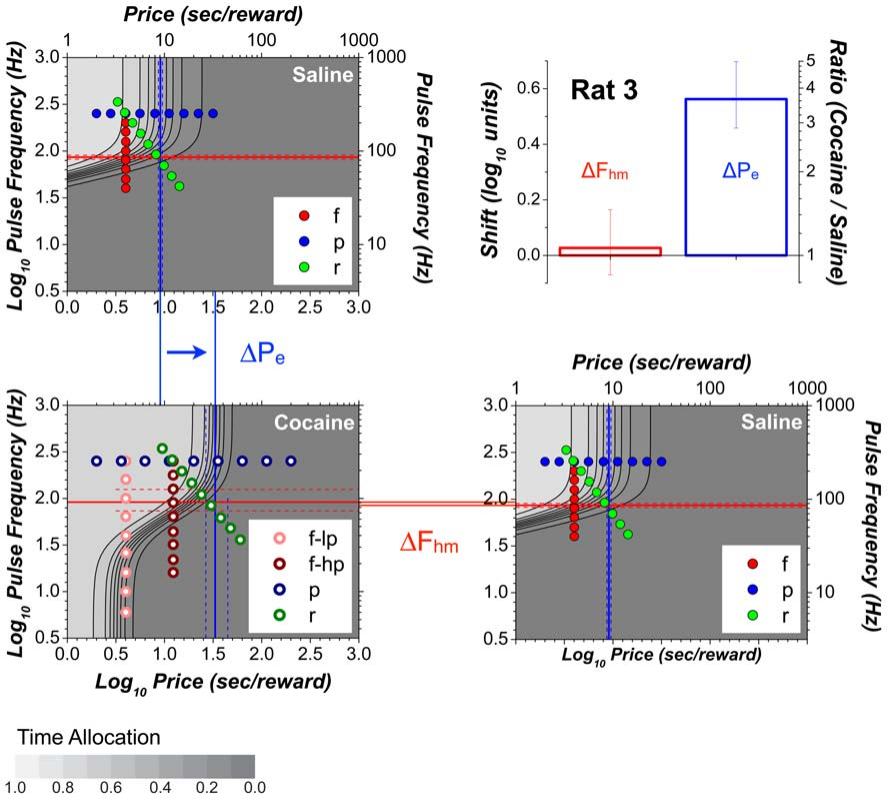
Shifts caused by cocaine in the position of the 3D structure fitted to the data from Rat 3. The data from Figure **9** are re-plotted as contour graphs so as to isolate and highlight the drug-induced shifts of the mountain (Δ*P_e_*, Δ*F_hm_*) along the price and frequency axes. The magnitudes of the shifts are contrasted in the bar graph. Error bars in the bar graph and dashed lines on the contour graphs are 95% confidence intervals. In the contour-map representation of the 7-parameter surface fitted to the data from the cocaine condition, note how the conditioned-reward parameter bends the contour lines downward at low frequencies and prices.

**Figure 11:**
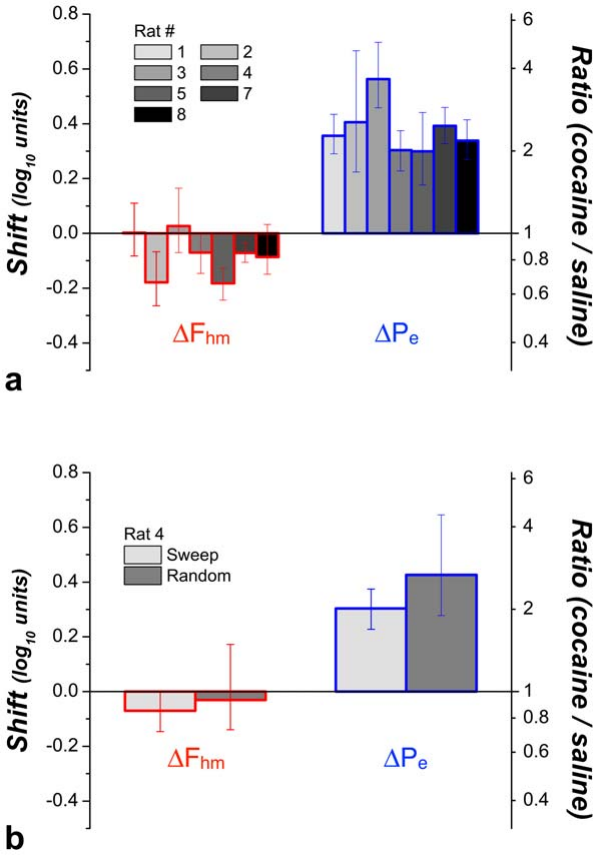
Shifts caused by cocaine in the position of the 3D structures fitted to the data from all subjects. **a**) Cocaine produced large and highly reliable shifts along the price axis (Δ*P_e_*) in all subjects. Shifts along the frequency axis (Δ*F_hm_*) are much smaller and do not always meet the statistical criterion. **b**) Comparison between the cocaine-induced shifts observed in the data from Rat 4 when the prices and pulse frequencies were sampled sequentially (“Sweep”) or randomly (“Random”). A similar pattern is observed in both cases. Error bars are 95% confidence intervals.

The cocaine-induced shifts in the location parameters for all seven rats are shown in the left-hand bar graph in Figure **11**. Note that reliable rightward displacements were observed along the price axis in all subjects (mean  = 0.38 log_10_ units); the rats were willing to pay opportunity costs 2–4 times higher under the influence of cocaine than during saline infusions. In contrast, displacements along the frequency axis were smaller (mean  = −0.08 log_10_ units), failed to meet the criterion for statistical reliability in 3/7 cases, and did so only marginally in a fourth (a result that did not hold up under random sampling, as shown in Figure **11b**).

All data in Figures **5** **,** **6** **,** **7** **,** **8** **,** **9** **,** **10** **,** **11a** were obtained by systematically incrementing or decrementing the value of one or both independent variables from trial to trial. Rat 4 was retested using an unpredictable order of presentation; the value of both the pulse frequency and the price were chosen randomly from vectors of values similar to those employed previously using the sweep procedure. As can be seen in Figure **11b**, the displacements of the mountain were similar regardless of whether the values of the independent variables were swept systematically over consecutive trials (light gray) or varied randomly (dark gray): whereas the 3D structure shifted substantially along the price axis, movements along the pulse-frequency axis were either unreliable (random condition) or barely discernable (sweep condition).

## Discussion

### The neurochemical basis of the observed shifts

Cocaine blocks the dopamine transporter, thereby increasing the magnitude and duration of spontaneously occurring dopamine transients in the nucleus accumbens terminal field [54]. In addition, cocaine increases the frequency of dopamine transients, despite the suppressive influence of somatodendritic autoreceptors. The increased frequency of transients may arise from blockade of voltage-gated sodium channels in local GABAergic interneurons [55], which releases dopaminergic cell bodies in the ventral tegmentum from inhibition. Together, these effects of cocaine contribute to the increase in the extracellular concentration of dopamine caused by the drug. Given the abundant evidence for a dopaminergic contribution to pursuit of BSR [56], it seems likely that the observed effects of cocaine arose, in large part, from drug-induced changes in dopaminergic signaling.

Cocaine also blocks the transporters for serotonin and norepinephrine [57]. The dominant influence of serotonin on pursuit of BSR appears to be opposite to the changes in reward pursuit reported here [58], [59], and thus, blockade of the serotonin transporter likely restricted the magnitude of these effects. In contrast, blockade of the norepinephrine transporter could well have contributed to the observed influence of cocaine. Neurons in the locus coeruleus and lateral tegmental A7 cluster show increased double labeling for tyrosine hydroxylase and the immediate early-gene product, Fos, following self-stimulation of MFB sites [60], suggesting that the activated neurons are noradrenergic. Injection of the α_1_ antagonist, terazosin, into the locus coeruleus produces rightward shifts in rate-frequency curves obtained from rats working for MFB stimulation [61]. Given evidence that activation of α_1_ receptors excites noradrenergic neurons in the locus coeruleus [62], the firing of these neurons would appear to contribute in some way to the pursuit of rewarding MFB stimulation. Cell bodies of noradrenergic neurons projecting to the VTA are located in the locus coeruleus [63], and electrical stimulation of the locus coeruleus has been shown to excite dopaminergic cell bodies in the VTA [64]. Thus, norepinephrine and dopamine may play synergistic roles in the enhanced pursuit of BSR produced by cocaine.

### Sensitivity versus gain changes in the BSR substrate

The 2D frequency-sweep data in Figure **5a** closely resemble results obtained previously using the curve-shift method to measure the effect of cocaine on performance for BSR: cocaine shifted the frequency-sweep curve leftward along the axis representing stimulation strength [65], [66], [67], [68]. Previous investigators have attributed such cocaine-induced enhancement of performance for BSR to a drug-induced increase in the sensitivity of the reward substrate [23], [24]. Before assessing how this proposal fares as an explanation of the data reported here and before discussing the 2D data in depth, it is important to explain exactly what is meant by the “sensitivity” of the BSR substrate and to contrast the meaning of this term with that of “gain.”

To express a change in the sensitivity of the BSR substrate in terms of the mountain model and the measurements reported here, we must distinguish among psychophysical, performance, and psychometric functions. A psychophysical function maps an objective stimulus variable, such as the luminance of a visual stimulus, into its subjective equivalent, such as brightness. For the value of a subjective variable to be inferred, it must be translated by a performance function into observable behavior (e.g., a verbal response such as “the test spot is twice as bright as the standard spot”). The embedding of a psychophysical function in a performance function yields a psychometric function, which has both a controllable objective input and an observable behavioral output. The 3D intensity-growth function at the left of Figure **3a** is a psychophysical function, the 3D behavioral-allocation function at the right of Figure **3a** is a performance function, and the curves in Figures **5** **,** **6** **,** as well as the mountain surface fitted to them, are psychometric functions.

**Figure 12:**
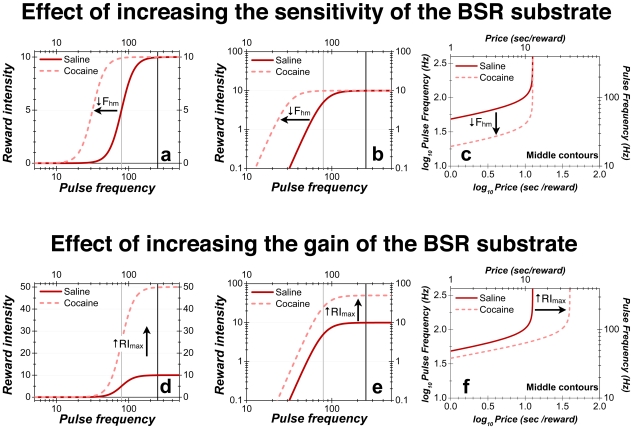
Sensitivity versus gain. The ***sensitivity*** of brain reward circuitry determines the stimulation strength required to produce a reward of a given intensity whereas the ***gain*** of the circuit determines the maximum intensity attainable. **a**) Simulated curves showing how a cocaine-induced increase in sensitivity shifts the intensity-growth function leftwards along the pulse-frequency axis. At the intersection of the black vertical line with the solid dark-red curve representing the saline condition, reward intensity is twice that at the intersection of this curve with the gray vertical line. In contrast, reward intensities are almost identical at the intersections of the two vertical lines with the dashed pink curve representing the cocaine condition. Thus, changes in sensitivity alter the relative values of rewards. **b**) Simulated data from panel **a** re-plotted in double logarithmic coordinates. **c**) The contour line halfway between the minimum and maximum time allocation. The cocaine-induced increase in sensitivity shifts the contour line downwards along the pulse-frequency axis. Note, that the form of the contour line mirrors the shape of the intensity-growth function in panel **b**. **d**) Simulated curves showing how a cocaine-induced increase in the gain of the BSR substrate produces equal proportional changes in all reward-intensity values. The intensity-growth function has been rescaled by the drug. Note that the ratios of reward intensities are equivalent at the intersections of the vertical lines with the solid dark-red curve for the saline condition (10∶5) and the dashed pink curve for the cocaine condition (50∶25). Thus, changes in gain alter absolute, but not relative, reward intensities. **e**) Simulated data from panel **d** re-plotted in double logarithmic coordinates. **f**) Effect of a gain increase on the position of the contour line halfway between the minimum and maximum time allocation. The contour line is shifted rightwards along the price axis.

Changes in ***sensitivity***, such as those that occur during light or dark adaptation (see below: “The broader significance of the distinction between sensitivity and gain”), reflect displacement of a psychophysical function along the axis representing stimulus strength. Let us substitute pulse frequency for luminance as the objective input to the psychophysical function and reward intensity for brightness as the subjective output. The result is shown as the solid dark-red curve in Figure **12a**. (The simulated curve plots the intensity-growth function for BSR [28], [41], [42], [69], as specified by Equations **1–3** in Text S1.) If, as proposed by several earlier investigators [23], [24], cocaine boosted the sensitivity of the BSR substrate, it would shift the psychophysical function leftwards, as shown by the position of the dashed pink curve in Figure **12a**. This effect is expressed in the mountain model as a decrease in the value of the *F_hm_* parameter. Figure **12b** re-plots the simulated curves in double logarithmic coordinates. In this representation, it can be seen readily that the form of the intensity-growth function is the same as that of the contour lines in Figures **8** **,** **10**. This is so because the input to the performance function in the mountain model (*U_B_* in Figure **3a**) is proportional to the ratio of reward intensity and price. Time allocation is constant along a contour line. As pulse frequency increases, driving reward intensity higher, the price must be increased in compensation so as to hold constant the ratio of reward intensity and price, and hence, time allocation (Fig. **12c**). However, as reward intensity approaches asymptote (*RI_max_* in Equation **3**, Text S1), further compensation is unnecessary, and the contour lines run vertically. Given that pulse frequency is represented on the abscissa of the intensity-growth function (Figs. **12a,b**) but on the ordinate of the contour graph (Fig. **12c**), increases in the sensitivity of the BSR substrate slide the psychophysical function leftwards (Figs. **12a,b**) and the contour lines downward (Fig. **12c**). These displacements are orthogonal to the principal effect of cocaine observed in this study: shifts of the mountain along the price axis. The proposed increase in sensitivity [**23]**, [24****] cannot account for such shifts.

In contrast to the failure of a sensitivity increase to explain the observed shifts along the price axis, an increase in the ***gain*** of the intensity-growth function (i.e., in the scaling of its output) can account readily for these results. A change in gain increases, by a common multiplicative factor, the reward intensity produced by each pulse frequency (Fig. **12d**). Thus, such a change is expressed in an additive vertical shift of the intensity-growth function in double logarithmic coordinates (Fig. **12e**) and is translated into a rightward shift of the mountain along the price axis, as illustrated by the contours in Figures **12f**. Such rightward shifts were seen in the data from all seven rats. Alternatively, or in addition, these shifts along the price axis may reflect other inputs to the performance function, such as subjective effort or opportunity costs, as discussed below.

### The inherent ambiguity of 2D representations

Despite the fact that the principal shifts in the 3D representation occurred along the price axis (Fig. **11**), the 2D projections of the mountain did shift along the pulse-frequency axis (e.g., Figs. **5a** **,** **6a**). Such a shift would also result from an increase in sensitivity. Thus, the 2D representations are ambiguous and that one cannot deduce the direction in which the 3D structure has shifted by examining a single 2D projection of this structure. Figure **2f** illustrates why this is so: It shows that changes in the position of a 2D psychometric function due to factors, such as gain changes, that operate at or beyond the output of the intensity-growth function (Figs. **2b** **,** **3c**) may be indistinguishable in practice from changes in position produced by altering the sensitivity of the reward substrate (Figs. **2c** **,** **3b**). The diagonal orientation of the face of the mountain causes the silhouette of the 3D structure to be displaced along the pulse-frequency axis as the mountain slides along the price axis (Fig. **2e,f**); similarly, sliding the mountain along the pulse frequency axis displaces the silhouette along the price axis (Movie S1, Movie S2). Thus, an observer confined to a 2D view of the plane defined by the time allocation and reward strength (e.g., pulse frequency) (Fig. **2f**) or price (Movie S1, Movie S2) cannot tell in which direction the mountain has moved. Such an observer cannot know whether a given manipulation altered a) the sensitivity of the reward substrate, b) any of the multiple factors that are brought to bear beyond the output of the intensity-growth function, or c) some combination thereof. In contrast, 3D measurement of the reward mountain readily distinguishes changes in sensitivity, which reflect changes in the value of *F_hm_*, from changes in willingness to pay, which reflects changes in the value of *P_e_*. Such 3D measurements reveal that cocaine produces large, consistent rightward shifts of the 3D structure along the price axis (increases in *P_e_*). Thus, earlier investigators were largely misled in attributing the shifts they observed in a 2D space to changes in sensitivity. Such an inference is based on the implicit assumption that shifts in a 2D psychometric function necessarily reflect analogous shifts in a particular psychophysical function embedded within it. **Figure 2** and Movies S1,S2 show why this assumption is untenable.

### Interpreting the 2D shifts

#### Application of the 3D information

The 3D representation makes clear the direction in which the mountain had been displaced by cocaine within the space defined by the strength and cost of BSR. This is particularly evident in the contour-graph comparisons (Figs. **9** **,** **11**). Once the direction of the shifts has been established in the 3D space, an unambiguous interpretation of the 2D graphs documenting each sweep type (Figs. **5** **,** **6**) can be provided. With the 3D information in hand, it can be seen that the leftward shifts shown in Figures **5a** **,** **6a** are due, largely (Figs. **5a** **,** **7** **,** **8**), or almost entirely (Figs. **6a** **,** **9** **,** **10**), to the rightward movement of the diagonally oriented face of the mountain along the price axis. Increasing the price by an amount roughly equal to the shift along the price axis (Fig. **5b**) restores the frequency-sweep curve obtained under the influence of cocaine to a position nearly identical to that of the curve obtained during saline administration.

The rightward shift of the price-sweep data in Figures **5c** and **6c** are an almost pure reflection of the shift of the mountain along the price axis. This is so because the price sweeps were carried out at pulse frequencies that produced near-maximal reward intensities, as indicated by the near-vertical orientation of the contour lines at their intersection with the trajectory of the price sweeps in Figures **8** **,** **10**. At such pulse frequencies, displacement of the mountain along the pulse-frequency axis cannot produce appreciable shifts of the 2D projection of the mountain along the price axis. This may appear to imply that the shift of the 3D structure along the price axis can be inferred from 2D price sweeps alone, but it does not. It is from the fit of the 3D surface to the data from all three sweep types that the intensity-growth function is derived; the parameters of this function must be known in order to determine whether the price sweeps were indeed carried out at reward-saturating pulse-frequencies. Thus, all three sweep types must be taken into account in order to measure the shift of the 3D structure along the price axis.

In both the saline and cocaine conditions, the trajectory of the radial sweep was positioned so as to pass through, or very near, the point defined by the two location parameters [*P_e_*, *F_hm_*]. When that condition is fully satisfied, the projection of the radial sweep in the plane defined by time allocation and pulse frequency (Figs. **5d** **,** **6d**) shows the shift of the mountain along the pulse-frequency axis, and the projection in the plane defined by time allocation and price (Figs. **5e** **,** **6e**) shows the shift along the price axis. Again, this may appear to imply that shifts in the 3D space can be inferred from the radial sweeps alone, but it does not. The 3D representation is required in order to determine whether the radial sweep indeed passed through [log_10_(*P_e_*), log_10_(*F_hm_*)]. When the trajectory of the radial sweep misses this point, as will usually be the case (at least by a small margin), the 2D projections of the radial sweep no longer provide a clean decomposition of the movement of the 3D structure. Again, all three sweep types must be taken into account.

#### An effect of cocaine on conditioned reward?

A feature of the 2D data that differs from prior reports is the substantially increased time allocation over the low-frequency portion of the frequency sweeps that was shown by some rats under the influence of cocaine (e.g., Figs. **6a** **,** **9** **,** **10**); this effect was greatest at the lower price. Such price-specific increases in responding for weak rewards may reflect cocaine-induced enhancement of conditioned reward [70]. Cues arising from withdrawal of the lever were present during the reward delivery. In the case of the price sweeps, pulse frequency was very high throughout, and thus, every withdrawal of the lever was accompanied by delivery of a powerful reward. The rat rarely satisfied the work requirement when reward intensity was low (over the low-frequency portions of frequency and radial sweeps). Thus, opportunities for appetitive Pavlovian conditioning between lever-withdrawal cues and delivery of strong rewards exceeded opportunities for extinction.

Conditioned rewards are preferentially enhanced by manipulations, such as stimulant administration, that boost dopamine tone [70]. If so, reward conditioned to the withdrawal of the lever may have been sufficiently high under the influence of cocaine to support responding for pulse frequencies too low on their own to produce a rewarding effect of substantial magnitude. It is striking that the mountain model can capture these effects gracefully via addition of a single parameter. This factor represents the pulse frequency required to produce a unconditioned reward equal in intensity to that produced by the conditioned stimulus (see Text S1). When the price is low, the payoff from the conjoint effects of low-frequency electrical stimulation and the conditioned reward is sufficient to support some reward-seeking behavior; however, when the value of the payoff-sensitivity exponent, *a*, is substantially greater than unity (as was always the case in this study), increasing the price quickly overshadows the contribution of the conditioned reward to time allocation, and frequency-sweep data obtained under the influence of cocaine resemble the form obtained in the saline condition provided that the price is sufficiently high (Figs. **6b** **,** **9**).

The predictions of the conditioned-reward version of the model are shown in Figures **6** **,** **9** **,** **10**; they are derived formally in Text S1, Equations 8,9.

We can now turn to the interpretation of the shifts revealed by the 3D perspective.

### Interpreting the 3D data

#### Shifts along the price axis

Figure **3** illustrates five different ways drugs could shift the mountain along the price axis; we view increases in gain and/or decreases in subjective costs as the most plausible interpretations of the data. The leftmost dashed gray arrow represents an increase in gain: an upward, drug-induced rescaling of the output of the intensity-growth function. Under such an influence, the reward intensity produced by each pulse frequency is multiplied by a constant greater than unity. This possibility is illustrated in Figures **12d–f**. The increases in gain shown in Figure 12**d,e** translate into rightward shifts of the mountain along the price axis (Fig. **12f**), the principal effect of cocaine that was observed in this study.

Cocaine-induced increases in sensitivity or gain move the 3D structure in orthogonal directions (Figs. **12c,f**). Nonetheless, these hypotheses bear a family resemblance. Like the related ideas proposed by Wise [71], they appeal to an increase in the intensity of BSR to account for the greater willingness of the subject to pay for a given stimulation train. These hypotheses stand in contrast to the remaining possibilities illustrated in Figure **3**, which concern inputs to the behavioral-allocation function other than reward intensity and which likely reflect the operation of brain circuitry other than that directly responsible for BSR.

A tenable alternative to the hypothesis that cocaine boosted gain in the BSR substrate is that the subjective value of the exertion required to hold down the lever was decreased by the drug. This notion is related closely to the view that dopamine tone modulates the proclivity of subjects to invest effort in the pursuit of reward [25], [26] and the vigor of consequent responding [72]. Thus, the proposals by Salamone and co-workers [25], [26], [73] and by Niv, Daw and Dayan [72] are compatible with the principal effects of cocaine reported here. Alternatively, or in addition, cocaine might reduce subjective opportunity costs in scalar fashion.

Although cocaine-induced reductions in subjective costs may well account for the observed shifts along the price axis, it is important to keep in mind that the face validity of this notion may be illusory. Just because the 3D structure moved along an axis representing opportunity cost, this does not mean that the subjective mapping of that cost (or effort cost) was changed by the drug. Shifts in psychometric curves do not necessarily imply corresponding shifts in a particular underlying psychophysical function; this is so because the performance function has multiple inputs that are combined in scalar fashion (Fig. **3a**). Thus, although the 3D structure was displaced by cocaine along an axis representing cost, the reason for this movement could have been a gain change in the BSR substrate and have had nothing to do with subjective estimates of either opportunity or effort costs. This point is of particular relevance to the interpretation of the effects of drugs and lesions on 2D measures of willingness to pay (e.g., progressive-ratio break points [74], [75]). Not only could changes in break point arise from any of the influences that shift the 3D structure along the price axis, they could also arise from changes in reward-substrate sensitivity, which shift the 3D structure along the reward-strength axis but its silhouette along the reward-cost axis (Movie S2).

Drug-induced modulation of subjective probabilities is another logically permissible interpretation of the price shifts, but we regard that view as far-fetched. The rewards in this experiment were delivered each and every time the response requirement was satisfied (i.e., p = 1), and subjective probability would have to have been increased substantially by cocaine in order to explain the data. This would require two-to-fourfold underestimation of reward probability in the saline condition, which seems highly unlikely.

Finally, shifts along the price axis could, in principle, result from a reduction in the payoff from alternate activities, such as grooming, resting, and exploring [76]. Although such an effect could have contributed, the magnitude of the observed shifts makes it rather unlikely that a decrease in the value of “everything else” is the sole cause. Willingness to pay increased by almost fourfold in Rat 3; in pilot testing with higher doses of cocaine, we have observed tenfold increases and higher. The likelihood of floor effects as the value of “everything else” approaches zero argues that a drug-induced reduction in the value of competing activities is unlikely to account for all or most of the observed shifts.

Future experiments that entail unambiguous inference of subjective costs should help distinguish between the plausible explanations of the shifts along the price axis. Highly non-linear functions likely map objective effort and opportunity costs [77] into subjective ones. If so, then the approach adopted here to measure lateral shifts of the non-linear intensity-growth function for BSR could be adapted to measure lateral shifts of the functions that determine subjective costs. That said, observation of a change in the product of several numbers (“payoff from BSR” in Figure **3a**) does not reveal which of the inputs to the calculation has been altered, and thus, changes in the gain of the BSR substrate (or scalar changes in subjective costs) cannot be isolated within the framework of the mountain model. However, if the circuitry underlying the intensity-growth function for BSR can be identified, then observation of its output would provide direct measurement of gain changes.

#### Shifts along the frequency axis

In addition to large shifts along the price axis, smaller shifts of the 3D surfaces along the pulse-frequency axis were seen in 4 cases. In one (Rat 4), this shift barely meets the criterion for statistical reliability and was not replicated when the rat was retested using random sampling of pulse frequencies and prices (Fig. **11b**). However, in two cases (Rats 2 and 5), the shifts along the pulse-frequency axis were substantial (−0.18 common logarithmic units), albeit much smaller than the corresponding shifts along the price axis (0.41 and 0.30 common logarithmic units, respectively).

Shifts along the pulse-frequency axis reflect drug actions prior to the output of the intensity-growth function, such as drug-induced increases in neurotransmitter release from directly stimulated neurons or modulating influences that magnify the impact of such release. It is not clear why shifts along the frequency axis were seen only in some subjects. That said, subtle changes in electrode placement that may be hard to discern in small samples are correlated with functional differences between MFB self-stimulation sites [78].

### Implications for the role of dopamine in brain stimulation reward

We have already shown that the principal and most consistent shifts observed in this study are orthogonal to those predicted by the hypothesis that cocaine-induced enhancement of dopaminergic neurotransmission boosts the sensitivity of the BSR substrate [23], [24]. We have shown further that the data on which this hypothesis is based can be explained by the way that shifts along the price axis alter the 2D projection of the mountain in the plane under consideration in the original work, the plane defined by behavioral performance and stimulation strength. Thus, the attribution of cocaine-induced enhancement of reward pursuit to increased sensitivity turns out to be based largely on an illusion that stems from viewing the data in a space with too few dimensions. We now consider some additional prior hypotheses concerning the mechanism underlying the enhanced pursuit of BSR produced by cocaine and the role played by dopaminergic neurons in this effect.

A particularly influential proposal was put forward by Wise in 1980 [71]:

“Drugs of abuse should enhance the effects of electrical stimulation, either bringing the reward system closer to its threshold for excitation, or reducing the number of neurons requiring electrophysiological activation by providing pharmacological activation of some portion of the critical neural pool.”

Both a reduction in threshold or in the number of neurons requiring electrophysiological activation entail a reduction in the aggregate rate of impulse flow required to produce a given level of reward intensity. Thus, this hypothesis is related closely to the view that psychomotor stimulants increase the sensitivity of the BSR substrate to electrical activation [23], [24], a view that cannot explain the observed shifts along the price axis. Nonetheless, this proposal also entails summation between activity of pharmacological origin that is independent of the electrical stimulation and impulse flow driven by the electrode. This is reminiscent of the conditioned-reward model proposed here and described formally in Text S1. What differs is that the stimulation-independent activity proposed in the conditioned-reward model is stimulus driven and phasic whereas Wise appears to have had in mind drug-induced changes in dopaminergic tone.

Wise's model and the increased-sensitivity hypothesis share with the increased-gain hypothesis a focus on the intensity of the reward signal induced by the electrical stimulation. A proposal by Moisan and Rompré [79] offers a way to account for such a gain increase in terms of known effects of cocaine on phasic dopaminergic signaling.

Moisan and Rompré [79] recorded the activity of trans-synaptically activated, midbrain dopamine neurons in response to stimulation of posterior mesencephalic reward sites. Similar trade-offs between the pulse frequency and the stimulation current (which determines the number of directly activated neurons) were obtained regardless of whether the dependent variable was the firing rate of dopamine neurons or the rate of lever pressing for rewarding stimulation. They concluded that a common mechanism is responsible: midbrain dopamine neurons spatially and temporally integrate input from the directly stimulated neurons responsible for BSR (Σ in Figure **3a**). (Alternatively, the dopamine neurons may lie downstream from the circuit that performs the spatiotemporal integration). An analogous arrangement may obtain in the case of rewarding MFB stimulation as well. Although dopaminergic fibers course through the MFB, they are fine and unmyelinated [80], rendering them difficult to activate with brief pulses of extracellular current delivered through macroelectrodes [81], [82], [83]. Given the currents, pulse durations, and electrode-tip exposures in the current study, relatively few dopaminergic fibers are likely to have been activated directly [2], [84]. However, rewarding MFB stimulation does produce robust activation of midbrain dopamine neurons [27], [85], [86], an effect attributed largely due to trans-synaptic activation [2], [54], [82]. If the dopamine neurons integrated input from the directly stimulated BSR substrate and translated the afferent impulse flow into a signal representing reward intensity, then cocaine-induced increases in the amplitude and duration of stimulation-induced dopamine transients [54], [85]would increase gain in the BSR substrate and shift the mountain along the price axis. This extension of Moisan and Rompré's hypothesis predicts that rats will self-stimulate for direct activation of dopamine neurons (e.g., by optogenetic means [87], [88]).

Shifts along the pulse-frequency axis reflect drug actions prior to the output of the intensity-growth function. To reconcile such shifts with our extension of Moisan and Rompré's hypothesis [79], cocaine would have to influence the input to midbrain dopamine neurons from afferents directly excited by the stimulation. For example, perhaps the sensitivity of the dopamine cells to these inputs is boosted by a cocaine-induced increase in noradrenergic drive [61]. Such an explanation would also have to include an account of why shifts along the pulse-frequency axis were seen only in some subjects.

In the model proposed by Moisan and Rompré [79], BSR depends on the phasic component of dopaminergic signaling. Hernandez and co-workers proposed an alternative “feedforward” model [27] in which tonic dopaminergic signaling gates transmission between non-dopaminergic neurons mediating BSR. If this model were modified to insert the dopaminergic gating signal at or beyond the output of the intensity-growth function, then it would provide a way for increased dopamine tone to boost gain in the neural circuitry subserving BSR. Increased dopamine tone could also play a role by decreasing subjective opportunity and/or effort costs.

Given the multiplicity of dopamine terminal fields [89] and the temporal multiplexing of dopaminergic signals [90], [91], there are many ways to map the multiple influences on reward pursuit depicted in Figure **3** onto dopamine signaling in different brain regions and different temporal components of dopaminergic neurotransmission. It will be necessary to carry out extensive experimentation, in independent-variable spaces of sufficient dimensionality, in order to discover which of the possible mappings is correct.

### Implications for the role of dopamine in the pursuit of natural rewards and drugs

Many prominent hypotheses concerning the role of dopamine in reward pursuit, such as the views related to allocation of effort and response vigor [25], [26], [72], [73], were developed to account for the effects of dopaminergic agents on performance maintained by natural rewards, such as food and water. The logic of the 3D approach applies to such experiments, which typically entail 2D analyses of behavior. To determine the stage(s) of processing at which neurochemical manipulations alter the pursuit of natural rewards, higher-dimensional spaces will have to be employed. Given the substantial evidence linking BSR to the rewarding effects of natural goal objects [92], [93], [94], there is good reason to suspect that the conclusions and hypotheses advanced here concerning the neurochemical basis of performance for BSR apply to the pursuit of natural rewards as well. However, such generalization does not yet have an empirical foundation, which requires extension of the 3D testing paradigm into the realm of natural rewards. To accomplish this, a variable such as sucrose concentration could be substituted for pulse frequency, and the methodology introduced by Conover, Shizgal and Woodside [92] employed.

Experiments on drug self-administration are often carried out by varying either the dose or cost of a drug. By substituting dose for pulse frequency, the 3D approach described here might be applied, in principle, to studying the effects of manipulations that alter pursuit of drugs. The 3D approach would allow changes in the sensitivity of the neural circuitry underlying the rewarding effect of a drug to be distinguished from the other determinants of reward pursuit.

### The broader significance of the distinction between sensitivity and gain

The distinction between changes in reward-system sensitivity and gain has broad implications, well beyond the study of intracranial self-stimulation. However, we know of no prior application of this distinction in the literature on reward, motivation, and affect. To make the case for the generality and significance of this distinction, we will first discuss its basis in an abstract manner and then provide examples concerning fundamental psychological processes.

Systems for encoding and processing information typically manifest non-linear behavior in response to extreme inputs. Noise imposes a limit on the weakest input that can be detected, and output ultimately saturates as the strength of the input grows. Thus, the input-output functions for such systems are typically sigmoidal in form (Figs. **12a,d**). Due to this S-like shape, some information will inevitably be lost; if the input is either too weak or too strong, it will fall within a range over which variation in input strength fails to alter the output. Optimal tuning of the input-output function requires that it be positioned so as to minimize the information loss. The slope of the sigmoidal input-output function is steepest over the middle portion, which means that the largest increment in output in response to a given increment in input occurs here. Thus, the encoding system will perform best when the steeply rising portion is centered over the middle of the range of inputs likely to be encountered.

In the example depicted in Figure **13.1a**, a hypothetical psychophysical function (the solid dark-red curve) maps objective luminance into subjective brightness for a moviegoer in a darkened theater. The hypothetical luminance distribution is shown below in blue. Note that the position of the psychophysical function is ideal for discriminating the luminance levels in the theater. Figure **13.1b** illustrates the problem that arises immediately after the moviegoer has stepped outside into daylight. The new luminance distribution is shown in light blue. Roughly the upper half of this distribution (cross-hatched) falls along the upper asymptote of the sigmoidal psychophysical function; the moviegoer will be unable to discriminate luminance levels in this range, and much potentially useful information will now be lost. What can his visual system do to adjust? Figure **13.1c** shows that a reduction in gain won't solve the moviegoer's problem. All non-zero brightness values will decrease by the same factor (dashed pink curve), but discrimination between luminances in the upper half of the outdoor distribution (cross-hatched area of the light-blue distribution) will be no better than before. In contrast, decreasing visual sensitivity solves the problem. This entails sliding the sigmoidal input-output function rightwards so that it again straddles the luminance distribution to which the moviegoer is exposed (dashed pink curve in Figure **13.1d**); loss of information about the outdoor scene has now been minimized by the adjustment in sensitivity. The cost of the adjustment in sensitivity is the information loss about more dimly lit scenes (cross-hatched area of the darker-blue distribution in Figure **13.1d**) that would occur, for example, if the moviegoer, now in a light-adapted state, darted back into the theater to retrieve an item inadvertently left behind.

**Figure 13:**
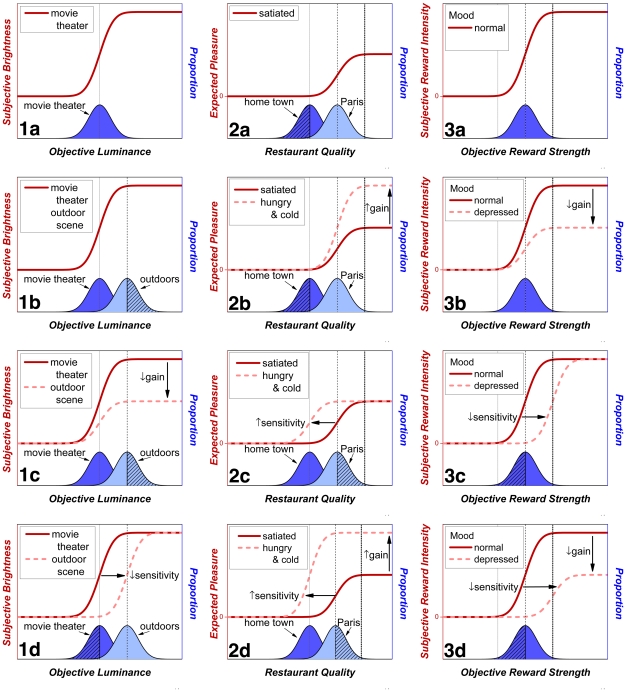
Three examples illustrating the broad applicability of the distinction between changes in gain and changes in sensitivity. The first example (left column) concerns light adaptation in the visual system, the second example (middle column) concerns changes in the prospective evaluation of meals due to changes in appetite and energy balance, and the third example (right column) concerns changes in the growth of reward intensity as a function of mood. (The spread of the luminance distribution in a real outdoor scene would almost surely be greater than in a darkened movie theater. The variances have been equated in the top row for simplicity of exposition.)

Let us now explore how the distinction between sensitivity and gain could apply in the realms of affect, motivation, and reward. Imagine that you derive your prospective intensity-growth function for the enjoyment of restaurant meals (solid dark-red curve in Figure **13.2a**) by visiting a range of establishments, both in your home town and in Paris. The output of the psychophysical function is your expected enjoyment of a meal at a given restaurant. On average, the restaurants in Paris are better than those in your home town, but the two distributions overlap. In a state of normal satiation, the psychophysical function is positioned to provide excellent discrimination between Parisian restaurants, at the cost of some information loss concerning the lower quality restaurants in your home town (cross-hatched region of the darker-blue distribution). The vertical lines designate three establishments: an average restaurant at home (thin leftmost line), an average restaurant in Paris (dashed middle line), and an outstanding restaurant in Paris (heavy dashed rightmost line). The prospective values of these three restaurants are distinguishable clearly. Now imagine that you are deprived of food for an entire day during which you hike a considerable distance over steep terrain in a cold driving rain. How might your intensity-growth function have changed? One possibility is depicted in Figure **13.2b**, which shows the effect of increasing gain. The value of a meal at any restaurant in the two distributions has been multiplied by two. Note that your relative preferences are unaltered: a meal at the average restaurant at home remains unattractive, and the expected enjoyment from a meal at the outstanding Parisian establishment outstrips the middle value assigned to the average Parisian restaurant. An alternate possibility is illustrated in Figure **13.2c**, which shows the effect of increasing sensitivity, thus shifting the psychophysical function leftwards. The maximum expected enjoyment of a meal has not changed, but relative preferences have been altered. When hungry, cold, and depleted of stored energy, you expect that the average meal at a home-town restaurant will be much more appetizing than when satiated, and it now seems worthy of consideration. A second consequence of the change in sensitivity is that discrimination fails over the upper end of the Parisian distribution (cross-hatched region in Figure **13.2c**). You are less choosy between high-end establishments, and both an average and outstanding Parisian restaurant are assigned the same maximal value. Thus, your discriminative capacity has been shifted toward the more mundane end of the range, expanding the number of acceptable choices. Figure **13.2d** shows the result of increasing both gain and sensitivity.

The set of example in the middle column of Figure **13** illustrates our ignorance about very basic issues in goal valuation. To our knowledge, no experiment carried out to date on food reward has distinguished between changes in gain and sensitivity. The data reported here concerning BSR provide a strong suggestion that different neural mechanisms likely subserve changes in gain and in sensitivity. In order to determine what these mechanisms are, a way must be found to distinguish changes in gain and sensitivity at the behavioral level. Extension of the mountain model to the realm of gustation could provide such a means and serve to isolate sensitivity changes.

A final example is in the realm of affect. Anhedonia is a defining feature of major depression [95]. How is reward processing altered by this disorder? Figure **13.3a** shows the growth of subjective reward intensity (solid dark-red curve) as a function of objective reward strength for an individual in a normal mood state. Figures **13.3b–d** show how depression would alter the growth of reward intensity by causing a decrease in gain (Fig. **13.3b**), a decrease in sensitivity (Fig. **13.3c**) or decreases in both gain and sensitivity (Fig. **13.3d**). The gain decrease blunts the values of all rewards without altering their relative values; no increase in objective reward strength can restore maximum reward intensity to the value attained in a normal mood state. In contrast, if it is sensitivity that is altered in depression, then a sufficiently strong reward can drive subjective reward intensity to its normal maximum, thus compensating partially for the influence of the affective disorder. However, some discriminatory capability is lost (cross-hatched region) due to the shift in the psychophysical function, and the indifference arising from the equivalence of weaker rewards could have serious consequences.

The examples in the right-hand column of Figure **13** show that changes in gain and sensitivity have different implications for the depressed individual. The mechanisms underlying such changes may well differ. Thus, determining how reward-growth functions change as a function of mood would appear important, both to achieving a better understanding of the nature of affective disorders and to developing improved remedies. Again, we know of no studies that have reported measurements appropriate to answering the basic question of how the mapping of objective reward strength into subjective reward intensity is altered in affective disorders and, more generally, by changes in mood states.

The discussion has been confined heretofore to two of the three essential parameters of a sigmoidal function: those specifying location (sensitivity) and scaling (gain). Also of interest is the remaining parameter, which determines the slope of the rising portion, (*g*, in the case of the mountain model). The larger the value of this parameter, the more discriminating the observer when processing inputs drawn from the central region of the input distribution. However, the cost of this high discrimination capacity is greater information loss at the extremes. The steeper the rise, the more narrow the region separating minimal and maximal responses. Thus, it would be useful indeed to be able to tune this parameter to match the dispersion (variance) of the input. When faced with the challenge of encoding a wide range of input values, decreasing the slope would minimize information loss; when faced with a narrow range of input values, a steep slope would maximize discriminability. These objectives are attained in photography by means of contrast adjustment. It would be very interesting to learn whether an equivalent process is at work in the realms of reward, motivation, and affect.

The examples discussed above illustrate the broader application of the distinction between sensitivity and gain, a distinction at the heart of the mountain model. The potential utility of this distinction in the study of reward, motivation, and affect speaks to the value of quantitative modeling. Such distinctions become clear once a formal model has been built, simulated, and applied but are often obscured when models are couched exclusively in verbal terms.

### The multidimensional basis of reward pursuit

The preceding examples explore a single component of the mountain model: the intensity-growth function for BSR. Implicitly included in the model are a set of additional psychophysical functions that map objective effort costs, opportunity costs, and probabilities into their subjective equivalents. A full generalization would also include the subjective mapping of delays in reward delivery (as well as the grouping of different goal objects into separate categories, such as different classes of nutrients, and the assignment of economic substitutability values [93], [96] to these categories). Like the intensity-growth function for BSR, the psychophysical mappings of effort costs, opportunity costs, probabilities and delays are almost certainly non-linear. Thus, it should prove possible to isolate contributions of these variables by applying the logic applied here to isolate changes in the sensitivity of the BSR substrate from other determinants of reward pursuit.

The mountain model and the above discussion of its future generalization show that a large multidimensional space is required to model reward seeking in a realistic manner. Limiting the number of dimensions explored simultaneously can render experimental results ambiguous with regard to the identity of the variable(s) responsible for the behavioral effects of a given manipulation (Fig. **2** and Movies S1,S2). By expanding the number of independent variables manipulated, applying a computational model, and exploiting both the remarkable stability of the intracranial self-stimulation paradigm and the high data-collection rates that can be achieved through its use, it has proved possible to provide some new answers to long-standing questions concerning the effects of an abused drug, cocaine, on reward seeking. That said, many additional questions remain to be addressed. Powerful methods for the specific activation or silencing of particular neural populations are emerging [97], [98], [99]. Increasingly sophisticated behavioral testing paradigms and computational models will be required in order to leverage these remarkable technological developments so as to better understand how the brain evaluates, selects, and pursues goals.

## Materials and Methods

### Subjects

Seven male Long-Evans rats (Charles-River, St. Constant, QC, Canada), weighing 300–350 g at the time of arrival, served as subjects. The experimental procedures were performed in accordance with the principles outlined by the Canadian Council on Animal Care. The protocol was approved by the Animal Research Ethics Committee of Concordia University (Protocol Number: AREC-2008-SHIZ).

### Surgery

Anesthesia was induced with Ketamine (10 mg/kg, i.p.) - Xylazine (100 mg/kg, i.p.) and maintained with Isoflurane. Stimulating electrodes were aimed bilaterally at the lateral hypothalamus (−2.8 AP, 1.7 ML, and −8.8 DV from the skull surface). The monopolar stainless-steel electrodes (0.25 mm diameter) were insulated with Formvar except for the region extending 0.5 mm from the tip. The anode consisted of two stainless steel screws fixed in the skull, around which the return wire was wrapped. A 5- to 7-day period was provided for post-surgical recuperation before the self-stimulation training began. Additional details are provided in a prior paper [38] as is the procedure for implanting the subcutaneous tubing used for continuous administration of cocaine or saline [100].

### Self-stimulation training and testing

As described in greater detail elsewhere [38], subjects were shaped to lever press for 0.5 s trains of cathodal, constant-current pulses, 0.1 ms in duration. The electrode that supported the most vigorous performance in the absence of motoric side effects was chosen for further testing. Once the rat pressed the lever consistently for currents between 250–400 µA, a curve relating time allocation to pulse frequency was obtained by varying the stimulation frequency across trials over a range that drove the number of rewards earned from maximal to minimal levels; at the beginning of this sequence of trials, the pulse frequency was set to the maximal value to be used and was then decreased successively from trial to trial. The series of trials conducted to obtain a time allocation versus pulse frequency curve is called a “frequency sweep.”

A “cumulative handling-time” schedule of reinforcement^47^ controlled the delivery of rewarding stimulation during data acquisition. Under this schedule, a reward is delivered when the cumulative time that the lever has been depressed reaches a value set by the experimenter (the “price” of the reward). The schedule is named with reference to the concept of handling time in behavioral ecology. “Handling” entails transformation of a prey object into consumable form (e.g., opening the shell of a nut or mollusc) and subsequent eating and digestion.

Depression of the lever was accompanied by illumination of the neighboring cue light. As soon as the response criterion was satisfied, the lever was retracted, and a stimulation train was delivered. After a 2-s delay, the lever was re-introduced into the cage, the cumulative timer was reset to zero, and the rat could resume working to obtain another reward.

Each trial consisted of a fixed time during which the price and pulse frequency parameters were held constant. The duration of each trial was sufficient to allow a rat that allocated all of its time to lever pressing to harvest 20 rewards. At the end of each trial and prior to the start of the next one, the lever retracted for 10 s, and the house light flashed. Two priming trains were delivered during the final 2 s of the inter-trial interval. The priming stimulation was held constant across trials and was delivered at a pulse frequency that had been shown previously to support vigorous responding; the remaining parameters were the same as those used during the test trials.

During frequency sweeps, the price of the reward was 4 s. This price was selected because at this and greater values, objective and subjective prices have been shown to correspond closely [77]. During price sweeps, the pulse frequency was set to the maximum value used during the frequency sweeps, and the price of the reward was increased successively from trial to trial. During radial sweeps, the pulse frequency was decreased successively, and the price was increased successively from trial to trial.

After stable performance was achieved in sessions that included all three sweep types, a second surgery was performed. The rats were anesthetized as described above, and a 24-cm loop of perforated Tygon® S-54-HL tubing (i.d.: 0.508 mm; o.d.: 1.52 mm; Saint-Gobain Performance Plastics, Akron, OH) was implanted subcutaneously. The Tygon tubing was attached to a short length of stainless-steel tubing, which was secured to the skull as described in a previous report [100].

#### Procedure

A sterile saline solution (0.9%) or a solution of cocaine hydrochloride, dissolved in sterile saline, adjusted to a pH of 7±0.1 by means of the addition of 0.1 M NaOH, was administered subcutaneously through the loop of porous subcutaneous tubing. The solutions were delivered at a constant rate (0.375 ml/h) by means of an external infusion pump (Harvard Syringe Pumps model 22, Harvard Apparatus, Saint Laurent, QC). The cocaine dose was either 1.75 mg/kg/h (Rats 1, 2, 3, 4, 7) or 3.5 mg/kg/h (Rats 5, 8). Two different doses were used because differential responding for low- and high-payoff stimulation trains delivered declined in the case of some subjects when the higher dose was administered during pilot testing.

During vehicle sessions, saline was infused subcutaneously. These sessions were run on Mondays and Thursdays and were composed of pairs of frequency, price, and radial sweeps. The position of each sweep during the session was randomized. During drug sessions, cocaine was infused subcutaneously. These sessions were run on Tuesdays and Fridays. In order to adequately sample the 3D structure acquired under the influence of the drug and to compare its position to the structure acquired during vehicle sessions, a second frequency sweep was added. One of the frequency sweeps acquired during the drug sessions (the “low-price” frequency sweep) was carried out at the same 4 s price as the frequency sweep in the vehicle sessions. The second frequency sweep acquired during the drug sessions (the “high-price” frequency sweep) was carried out at a higher price chosen to offset the influence of the drug. The addition of a second frequency sweep, and the need to test higher prices in the drug sessions made it unfeasible to include all four sweep types in a single drug session. Thus, each drug session consisted of a subset of the four sweep types, and multiple drug sessions were required to obtain enough data to fit the 3D structure.

Rat 4 was retested using randomly sampled values of the pulse frequency and the price. Three matrices were constructed with the same structure as those specifying the frequency, price, and radial sweeps run previously. Each matrix consisted of a nine-element column of pulse frequencies and a nine-element column of prices. The pulse frequencies were ∼0.11–0.12 common logarithmic units higher than those tested in the sweep phase, but the prices tested were the same. For the cocaine condition, the price in the matrix modeled on the frequency sweep was the higher of the two values tested in the sweep procedure. Trials were run in triads, with the experimental trial bracketed by trials run with fixed parameters that produced either a high or low payoff. During the lead trial of each triad, the price was always 1 s, and the pulse frequency was the highest value that the rat could tolerate without signs of aversion or uncontrolled stimulation-induced movement. During the trailing trial of each triad, the price was again 1 s, but the pulse frequency was too low to support operant responding. During the middle, experiment trial, the matrix and the row from which the pulse frequency and price were drawn were determined randomly.

Whether sweeps or random sampling of pulse frequencies and prices were used, the self-stimulation tests began 2 h after the start of the cocaine or saline infusion. The first determination of the time-allocation-versus-frequency curve was considered a warm-up and not included in the analysis. The collection of the behavioral data was restricted to the period when the cocaine-induced elevation in DA concentration had been shown to be stable for a dose of 10 mg/kg/h [100] or for a dose of 1.75 mg/kg/h [101]. After the first week of experimentation, a preliminary fit of the mountain model to the data was performed, and the results were used to adjust the tested values of pulse frequency and price so as to optimize sampling. The new values were selected so as to accommodate the drug-induced displacement of the 3D structure and to select the price for the high-price frequency sweep that was included in the drug condition. The price in question was chosen to offset the drug effect so that the time-allocation versus pulse frequency plot for the high-price frequency sweep carried out in the cocaine condition would overlap the plot obtained at the lower price employed in the saline condition.

### Data analysis

The 3D model was fitted separately to the data from the vehicle and drug sessions using the non-linear least-squares routine (lsqnonlin.m) in the MATLAB Optimization Toolbox (The Mathworks, Natick, MA) and an approach based on resampling [52]. The fitting procedures described below returned estimates of all model parameters for each dataset along with 95% confidence intervals. A shift in a location parameter was deemed statistically reliable when the 95% confidence interval around the difference between the values for the cocaine and saline conditions failed to include zero.

Graphs of the fitted surfaces were plotted using Origin v8.0 (OriginLab Corporation, Northampton, MA) as were the contour graphs of the 3D structure. Also plotted, for each sweep type, were the time-allocation means, their associated 95% confidence intervals, and the 2D projections of the fitted surface. The 3D images in Figures 1,2 were prepared using Mathematica v7.01 (Wolfram Research, Champaign, IL).

#### Surface fitting

One objective of the fitting approach was to obtain an unbiased measure of the dispersion of the parameter estimates. We adopted a resampling strategy [52] to achieve this. By sampling randomly with replacement from the data, we obtain multiple samples. The 3D model was fit to each of these samples, thus allowing us to compute empirically derived 95% confidence intervals around each of the model parameters as well as around the differences between the estimates of each parameter for the vehicle and drug conditions: The 95% confidence interval around each estimate was defined as the region excluding the lowest 2.5% and highest 2.5% of the estimates. The resampling strategy and the empirically derived confidence intervals it generates allow us to avoid making unrealistic assumptions about a lack of correlation between the estimates of the different parameters and about the normality of the parameter-estimate distributions.

**Figure 14:**
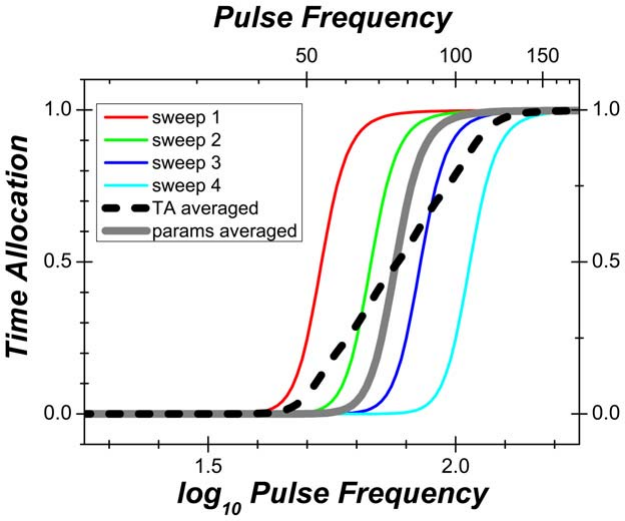
Appropriate and inappropriate ways to average frequency-sweep curves. Averaging the parameters of the individual curves (thick gray curve) yeilds an averaged curve with a representative slope whereas averaging the time-allocation values produces an averaged curve with unrepresentative shallow slope.

Another objective of the fitting approach was to avoid the bias in slope estimates that is introduced by conventional across-session averaging. This problem can be seen readily in a simplified 2D example. Imagine that time-allocation versus pulse frequency curves are obtained repeatedly across multiple testing sessions (thin colored curves in Figure **14**). Noise in the determinants of the position parameter displaces these curves leftwards or rightwards. If a curve is constructed from the mean of the time-allocation estimates, (heavy dashed curve) its slope will be more gradual than those of any of the individual curves it is supposed to represent. This problem can be circumvented by separately fitting an appropriate model to each of the curves and then averaging the parameter estimates instead of the data points [27]; the resulting (heavy gray) curve has the appropriate slope and is positioned in the center of the cluster. Generalized to 3D, this is what was done to avoid bias in the estimates of the two parameters (*a*, *g*) that determine the slopes of the 3D structure along the price and pulse-frequency axes.

The resampling strategy was adapted to conform to the different structures of the vehicle and drug sessions. Each vehicle session consisted of two complete sets of sweeps (frequency, price, and radial). Thus, these data were resampled by session. Consider the case of Rat 8. Each of the 1000 sets of resampled data consisted of data from nine sessions chosen at random and with replacement from the nine vehicle sessions that were run. Thus, the list of sessions included in a typical resampled data set could consist of the session numbers (1, 2, 2, 4, 6, 7, 7, 8, 9); the list of sessions included in another resampled data set could be (2, 3, 4, 5, 5, 6, 8, 9, 9).

Both the 6- and 7-parameter 3D models (Supplementary Equations) were fit in two different ways to the resampled data. The “location-specific” approach is aimed at optimizing the accuracy of the slope-parameter estimates; accuracy is crucial because the slope parameters interact with the location parameters. The model is fit separately to the data from each session in the resampled list, with the two location parameters (*F_hm_*, *P_e_*) free to vary across sessions and common values of the remaining parameters (*a*, *g*, *TA_max_*, *TA_min_*). This approach captures across-session drift in the location parameters while avoiding the explosion in the number of free parameters and the consequent increased uncertainty in their estimates that would have obtained had all six parameters been free to vary across sessions. For example, in the case of the 9-session dataset obtained from Rat 8, a 22-parameter model was fit (one estimate per session for each of the two location parameters plus single estimates of the remaining four parameters). Had all six parameters been free to vary across sessions, a 54-parameter model would have been fit.

The second, “all common,” approach entails fitting the 3D model to the pooled data from all of the sessions in the resampled list. Thus, only 6 parameters are estimated (or 7, in the case of the conditioned-reward model in Text S1). If the across-session drift in the location-parameter estimates is small in comparison to other noise sources, this second method may be preferable to the first due to the greatly reduced number of parameters.

The remainder of the procedure is common to both approaches. On each iteration, the parameter estimates for the resampled data were averaged across the resampled sessions. Thus, in the case of Rat 8, the location-specific approach yielded nine estimates of each of the location parameters (*F_hm_*, *P_e_*), and these were averaged on each iteration to yield a single estimate for each parameter per iteration. One thousand iterations were performed, and the means of the 1000 resulting parameter estimates and the associated 95% confidence intervals were then computed.

Due to the increased time required to test higher prices in the drug condition and due to the addition of a second frequency sweep in that condition, all sweep types were not run in a single session. Therefore, it was not feasible to resample the data by session. Instead, the data were resampled by sweep. One sweep of each type was sampled at random, with replacement, from the pool of all sweeps of that type so as to create a dataset to which the 3D model could be fit; the number of datasets so constructed equaled the number of sessions run in the drug condition. In some cases, the different pools (low-price frequency sweeps, high-price frequency sweeps, price sweeps, and radial sweeps) contained different numbers of sweeps. This resulted in differential sampling of the pools, with those containing fewer sweeps sampled more heavily than those containing more sweeps. To compensate, the contribution of each sweep type to the fit was weighted by the number of sweeps of that type. The remainder of the fitting procedure was the same as for the vehicle data.

Calculation of the AIC [53] allowed us to determine which fitting method worked best, i.e., whether the additional parameters associated with the location-specific approach “pulled their weight.” The AIC achieves this by assigning a penalty for each added parameter. We determined the AIC for what we call the “primary” fit of the model. This is the fit to the raw data (i.e., carried out in the absence of resampling). In the example provided above (results from Rat 8), the dataset thus consisted of the results from sessions 1 through 9.

#### Estimating shifts in the values of the location parameters

The parameters obtained from the most successful fitting method, as determined from the AIC scores, were used subsequently to estimate the shifts produced by cocaine in the value of the location parameters, *F_hm_* and *P_e_*. For each dataset, a 1000-element vector was obtained by subtracting the 1000 estimates of a given location parameter for the vehicle condition from the corresponding estimate for the cocaine condition. The shifts reported in the Results section and shown in Figures **8** **,** **10** **,** **11** are the means of the resulting difference vectors; the error bars are the empirically calculated 95% confidence intervals (the ranges between the 26^th^ and 975^th^ elements in the sorted difference vectors).

### Histology

After the completion of the experiment, a lethal dose of sodium pentobarbital was administered. A 1 mA anodal current was passed through the stimulating electrode for 15 s to deposit iron ions at the site of the electrode tip. The animals were then perfused intracardially with 0.9% sodium chloride, followed by a formalin-Prussian Blue solution (10% formalin, 3% potassium ferricyanide, 3% potassium ferrocyanide, and 0.5% trichloroacetic acid) that forms a blue reaction with the iron deposited at the tip of the electrode. Then, the rats were decapitated and their brains were removed and fixed with 10% formalin solution for at least 7 days. Coronal sections, 30 µm thick, were cut with a cryostat (Thermo Scientific) and stained subsequently using the formol-thionin technique. Tip locations were determined microscopically at low magnification with reference to the stereotaxic atlas of Paxinos and Watson [51].

## Supporting Information

Movie S1 **An overview of the two movies is provided in Text S2.**
Movie S1 consists of four short segments. It pauses after each one and resumes following a mouse click within the movie window. Pressing the back arrow on the keyboard will play the current segment backwards, returning to the beginning of the segment.
Initial condition: The surface of the mountain is denoted by a purple mesh in panel **A**. The outline of the reward mountain in the plane defined by time allocation and pulse frequency (the variable that controls reward strength) is shown in yellow.
Click 1: The mountain slides along the pulse-frequency axis (as denoted by the red arrow). The initial position of the outline is shown in black and the final position in yellow. The space between the black and yellow outlines is colored orange under the purple mesh.
Click 2: The mountain returns to its initial position. A little green figure (“Flatman;” Shutterstock Images LLC) drops in from above and stands viewing the mountain from the pulse-frequency axis. This observer perceives the world in only two dimensions. Thus, from Flatman's viewpoint, the price dimension does not exist. Flatman's 2D view is shown in the green bubble (panel **B**) as a conventional graph of performance (time allocation) versus a reward-strength variable (pulse frequency). This graph is analogous to a plot of data from a conventional “curve-shift” experiment [35], [36], [37].
Click 3: The mountain returns to its initial position and is displayed in 3D in panel **C**. It then slides along the price axis (as denoted by the blue arrow), moving in an orthogonal direction to the displacement that was shown in panel **A** following clicks 1 and 2. The face of the mountain includes a diagonally oriented segment. Thus, as the mountain slides along the price axis, its outline (dashed yellow curve) is displaced leftwards along the pulse-frequency axis.
Click 4: The mountain returns to its original position in panel **C**. Flatman then reappears and the mountain again slides along the price axis. What Flatman sees from his 2D viewpoint along the pulse-frequency axis is shown inside the green bubble in panel **D**. Note that the two orthogonal displacements of the mountain are clearly distinguishable in the 3D views (panels **A,C**) but are indistinguishable in Flatman's conventional 2D view (panels **B,D**).
(MOV)

Movie S2 **An overview of the two movies is provided in Text S2.**
Movie S2 consists of four short segments. It pauses after each one and resumes following a mouse click within the movie window. Pressing the back arrow on the keyboard will play the current segment backwards, returning to the beginning of the segment.
Initial condition: The surface of the mountain is denoted by a purple mesh in panel **A**. The outline of the reward mountain in the plane defined by time allocation and price is shown in yellow.
Click 1: The mountain slides along the price axis (as denoted by the blue arrow). The initial position of the outline is shown in black and the final position in yellow. The space between the black and yellow outlines is colored blue under the purple mesh.
Click 2: The mountain returns to its initial position. A little green figure (“Flatman;” Shutterstock Images LLC) drops in from above and stands viewing the mountain from the price axis. This observer perceives the world in only two dimensions. Thus, from Flatman's viewpoint, the pulse-frequency dimension does not exist. Flatman's 2D view is shown in the green bubble (panel **B**) as a conventional graph of performance (time allocation) versus price (required work time to obtain a reward). This graph is analogous to a plot of data obtained in a progressive-ratio experiment [74].
Click 3: The mountain returns to its initial position and is displayed in 3D in panel **C**. It then slides along the pulse-frequency axis (as denoted by the red arrow), moving in an orthogonal direction to the displacement that was shown in panel **A** following clicks 1 and 2. The face of the mountain includes a diagonally oriented segment. Thus, as the mountain slides along the price axis, its outline (dashed yellow curve) is displaced rightwards along the price axis.
Click 4: The mountain returns to its original position in panel **C**. Flatman then reappears and the mountain again slides along the pulse-frequency axis. What Flatman sees from his 2D viewpoint along the price axis is shown inside the green bubble in panel **D**. Note that the two orthogonal displacements of the mountain are clearly distinguishable in the 3D views (panels **A,C**) but are indistinguishable in the conventional 2D view (panels **B,D**).
(MOV)

Text S1 (DOC)

Text S2 (DOC)
