# Supplementary material for: At What Stage of Neural Processing Does Cocaine Act to Boost Pursuit of Rewards?
Source: PLoS One. 2010 Nov 30;5(11):e15081. doi: 10.1371/journal.pone.0015081 (PMC2994896; doi:10.1371/journal.pone.0015081)
Supplement: Text S1 — (DOC) [file pone.0015081.s003.doc]

## Derivation of the Reward-Mountain Model

### The 6-parameter variant of the mountain model

This version of the mountain model is closely related to the one presented by Arvanitogiannis and Shizgal [1] but has been modified to accommodate the fixed cumulative handling-time schedule of reinforcement [2] employed in the current study. Each directly stimulated neuron (left side of Fig. **3a**) is assumed to fire once per stimulation pulse. The current is held constant, which fixes the boundary of the effective stimulation field and the number of directly stimulated neurons. Hence, the aggregate spike rate is directly proportional to the pulse frequency. The intensity-growth function is described by Equations 1 [1,3] and 2 [4]:

(1)

where D = duration of the stimulation train

*F* = pulse frequency

*F_hm_* = pulse frequency that produces a half-maximal reward

intensity

*g* = the intensity-growth exponent,

and *RI_rel_* = relative reward intensity, which varies from 0 to 1

(2)

where C = the chronaxie of the strength-duration function for trains: the duration at which *F_hm_* is twice

and = the rheobasic (asymptotic) value of *F_hm_*, i.e., for a train of

infinite duration

The *F_hmr_* parameter determines the ***sensitivity*** of the BSR substrate to the electrical stimulation. Equation 1 describes the red curve in the graph of intensity-growth function at the left of Figure **3a** whereas Equation 2 describes the dark cyan curve in that graph. This functional form of Equation 1 was inspired by the results of the experiment of Gallistel and co-workers [3,5].

The scale, or ***gain***, of the intensity-growth function is determined as follows:

(3)

where *RI_max_* = maximum reward intensity (the gain parameter)

Evaluation of BSR manifests a property call “duration neglect” [4]. A parsimonious way to account for this property is to pass the output of the intensity-growth function through a peak detector [4,6] en route to memory so that only the maximum value registered during the stimulation train is stored. Hence:

(4)

where *RI_peak_* = peak reward intensity registered during the stimulation train

By analogy to traditional accounts, such as the generalized matching law [7,8,9], the payoff from BSR represents the scalar combination of subjective reward quality, likelihood, and cost. Specifically, the stored values of peak subjective reward intensity (the proxy for reward quality), subjective probability, subjective opportunity cost (price) and the subjective rate of exertion (effort cost) are combined as follows [1]:

(5)

where *p* = subjective probability that BSR will be delivered once the

work requirement has been satisfied

*P* = price (subjective opportunity cost)

*U_B_* = net payoff from BSR

and 1+*ξ* = subjective rate of exertion required to hold down the lever

The addition of 1 in the denominator prevents explosive growth of *U_B_* as *ξ* becomes very small.

An expression related to McDowell’s generalization [10] of Hernnstein’s single-operant matching law [11,12] is used to allocate behavior between pursuit of BSR and engagement in alternate activities, such as resting, grooming, and exploring:

(6)

where *a* = the payoff-sensitivity exponent

*TA_max_* = maximum time allocation

*TA_min_* = minimum time allocation

and *U_E_* = payoff from alternate activities

The *TA_max_* parameter is required because physical constraints and side effects of the stimulation may prevent the rat from keeping the lever depressed during the entire time it is extended, even when payoff from BSR is very high. Similarly, the *TA_min_* parameter is required because the rat usually spends some time sampling the lever, even when payoff is low.

In the McDowell equation [10], the exponent, *a*, captures deviation from matching (over- or under-matching). Given that the schedule of reinforcement employed here shares the property of ratio schedules that the number of rewards earned is directly proportional to time worked, over-matching is predicted, and, as expected, the value of *a* always exceeded unity. Conover and Shizgal [13] have interpreted *a* in terms of the substitutability of BSR with alternate sources of reward, such engagement in resting, grooming, and exploring. On that view, greater substitutability is reflected in higher values of *a*. The slope of the mountain profile along the price axis is determined by the value of *a* whereas the slope of the profile along the pulse-frequency axis is determined by the values of both *a* and *g*.

Combining Equations 1, 2-6 yields the 6-parameter expression that was fitted to the 3D data:

(7)

where , the price at which time allocation for a maximally

intense reward falls midway between *TA_max_* and *TA_min_*.

### The 7-parameter “conditioned-reward” variant of the mountain model

To account for data such as those in Figures **6,9,10**, the intensity-growth function was generalized to include a term reflecting conditioned reward, a reward associated with lever-related stimuli and/or the act of holding down the lever. Thus, Equation 1 has been extended as follows:

(8)

where *F_CR_* = the contribution of the conditioned reward, expressed in

terms of the pulse frequency required to produce an

unconditioned reward of equal intensity to the

conditioned reward

Equation 8 treats the conditioned reward and the input from the electrode in an equivalent manner. The functional form constrains the interaction of the conditioned and unconditioned rewards to an early stage of processing. *RI_rel_* has the same maximum value in Equations 1 and 8. Thus, *RI_max_* is unchanged by the generalization of the intensity-growth equation to accommodate the rewarding impact of stimuli associated with delivery of the stimulation train. This feature confines the effect of the conditioned reward to the profile of the mountain along the pulse-frequency axis (Figs. **6,9,10**).

Substitution of Equation 8 for Equation 1 generalizes Equation 7 to read:

(9)

Equation 9 is the 7-parameter mountain model that was fitted to the data, in addition to the 6-parameter model specified by Equation 7. Calculation of the Akaike Information Criterion [14] for the 6- and 7-parameter models was used to determine which is best.

## References

1. Arvanitogiannis A, Shizgal P (2008) The reinforcement mountain: allocation of behavior as a function of the rate and intensity of rewarding brain stimulation. Behav Neurosci 122: 1126-1138.

2. Breton YA, Marcus JC, Shizgal P (2009) Rattus Psychologicus: construction of preferences by self-stimulating rats. Behav Brain Res 202: 77-91.

3. Simmons JM, Gallistel CR (1994) Saturation of subjective reward magnitude as a function of current and pulse frequency. Behavioral Neuroscience 108: 151-160.

4. Sonnenschein B, Conover K, Shizgal P (2003) Growth of brain stimulation reward as a function of duration and stimulation strength. Behav Neurosci 117: 978-994.

5. Leon M, Gallistel CR (1992) The function relating the subjective magnitude of brain stimulation reward to stimulation strength varies with site of stimulation. Behavioural Brain Research 52: 183-193.

6. Gallistel CR (1978) Self-stimulation in the rat: Quantitative characteristics of the reward pathway. Journal of Comparative and Physiological Psychology 92: 977-998.

7. Baum WM, Rachlin HC (1969) Choice as time allocation. Journal of the Experimental Analysis of Behavior 12: 861-874.

8. Killeen P (1972) The matching law. J Exp Anal Behav 17: 489-495.

9. Miller HL (1976) Matching-based hedonic scaling in the pigeon. Journal of the Experimental Analysis of Behavior 26: 335-347.

10. McDowell JJ (2005) On the classic and modern theories of matching. J Exp Anal Behav 84: 111-127.

11. Herrnstein RJ (1970) On the law of effect. Journal of the Experimental Analysis of Behavior 13: 243-266.

12. Herrnstein RJ (1974) Formal properties of the matching law. Journal of the Experimental Analysis of Behavior 21: 159-164.

13. Conover KL, Shizgal P (2005) Employing labor supply theory to measure the reward value of electrical brain stimulation. Games and Economic Behavior 52: 283-304.

14. Akaike H (1974) A new look at the statistical model identification. IEEE transactions on automatic control 19: 716-723.
