# Supplementary material for: At What Stage of Neural Processing Does Cocaine Act to Boost Pursuit of Rewards?
Source: PLoS One. 2010 Nov 30;5(11):e15081. doi: 10.1371/journal.pone.0015081 (PMC2994896; doi:10.1371/journal.pone.0015081)
Supplement: Text S2 — (DOC) [file pone.0015081.s004.doc]

## Overview of Movie S1 and Movie S2

The two movies illustrate the ambiguity inherent in two-dimensional (2D) views of data from experiments on reward seeking. Performance in such experiments depends both on the strength and cost of rewards. Nonetheless, these independent variables are manipulated singly in typical experiments. The results are viewed typically in a 2D space, with the vigor of performance represented on the ordinate and the independent variable (strength or cost of reward) on the abscissa.

Shizgal’s “reward-mountain” model (Text S1) expands the space for visualizing data from experiments on reward-seeking. This model proposes a specific form for the function that maps the strength and cost of reward into performance. It has been formulated to map the strength and cost of rewarding electrical brain stimulation into the proportion of trial time allocated to reward pursuit; work time required to obtain a reward serves as the cost variable. In principle, this model could be generalized to the pursuit of natural rewards, such as food and water, to other cost variables, such as required physical exertion, and to other measures of performance, such as response rate.

The movies display predictions of the reward-mountain model, providing both 2D and 3D views. Whereas the direction in which the mountain moves is evident in the 3D views, orthogonal movements can be indistinguishable in the 2D views. According to the reward-mountain model, the direction of displacement reflects the stage of neural processing at which manipulations such as drug administration, lesions, and physiological challenges act to alter reward pursuit. Thus, it is important to resolve the ambiguity inherent in the conventional 2D views, which cannot determine the direction of displacement. The reward-mountain model and the associated behavioral-sampling methods achieve this.
